# Repression by the H-NS/YmoA histone-like protein complex enables IscR dependent regulation of the *Yersinia* T3SS

**DOI:** 10.1371/journal.pgen.1010321

**Published:** 2022-07-28

**Authors:** David Balderas, Mané Ohanyan, Pablo A. Alvarez, Erin Mettert, Natasha Tanner, Patricia J. Kiley, Victoria Auerbuch

**Affiliations:** 1 Department of Microbiology and Environmental Toxicology, University of California, Santa Cruz, California, United States of America; 2 Department of Biomolecular Chemistry, University of Wisconsin-Madison, Madison, Wisconsin, United States of America; Michigan State University, UNITED STATES

## Abstract

The type III secretion system (T3SS) is an appendage used by many bacterial pathogens, such as pathogenic *Yersinia*, to subvert host defenses. However, because the T3SS is energetically costly and immunogenic, it must be tightly regulated in response to environmental cues to enable survival in the host. Here we show that expression of the *Yersinia* Ysc T3SS master regulator, LcrF, is orchestrated by the opposing activities of the repressive H-NS/YmoA histone-like protein complex and induction by the iron and oxygen-regulated IscR transcription factor. While deletion of *iscR* or *ymoA* has been shown to decrease and increase LcrF expression and type III secretion, respectively, the role of H-NS in this system has not been definitively established because *hns* is an essential gene in *Yersinia*. Using CRISPRi knockdown of *hns*, we show that *hns* depletion causes derepression of *lcrF*. Furthermore, we find that while YmoA is dispensable for H-NS binding to the *lcrF* promoter, YmoA binding to H-NS is important for H-NS repressive activity. We bioinformatically identified three H-NS binding regions within the *lcrF* promoter and demonstrate binding of H-NS to these sites *in vivo* using chromatin immunoprecipitation. Using promoter truncation and binding site mutation analysis, we show that two of these H-NS binding regions are important for H-NS/YmoA-mediated repression of the *lcrF* promoter. Surprisingly, we find that IscR is dispensable for *lcrF* transcription in the absence of H-NS/YmoA. Indeed, IscR-dependent regulation of LcrF and type III secretion in response to changes in oxygen, such as those *Yersinia* is predicted to experience during host infection, only occurs in the presence of an H-NS/YmoA complex. These data suggest that, in the presence of host tissue cues that drive sufficient IscR expression, IscR can act as a roadblock to H-NS/YmoA-dependent repression of RNA polymerase at the *lcrF* promoter to turn on T3SS expression.

## Author summary

In facultative pathogens, horizontally-acquired virulence factors must be integrated into existing regulatory networks that respond to environmental cues. The Ysc type III secretion system (T3SS) is encoded on a virulence plasmid carried by the plague agent *Yersinia pestis* and the closely related enteropathogen *Yersinia pseudotuberculosis*. H-NS is a histone-like protein suggested to repress horizontally-transferred virulence genes. Here we show that in *Y*. *pseudotuberculosis*, H-NS and its binding partner YmoA facilitate oxygen-dependent regulation of the Ysc T3SS by the transcription factor IscR. As oxygen availability is low in the intestine where the T3SS is not required for *Yersinia* colonization, and higher in extraintestinal tissue where the T3SS is critical for virulence, these data suggest that the Ysc T3SS was integrated into the pre-existing H-NS and IscR regulatory networks to optimize bacterial pathogenesis.

## Introduction

Virulence factors are critical components that allow pathogens to establish or sustain infections within a given host. One common bacterial virulence factor is a needle-like apparatus, known as the type III secretion system (T3SS) [1,2]. Enteropathogenic *Yersinia pseudotuberculosis* is one of three human pathogenic *Yersinia* spp. that use the T3SS to inject effector proteins into host cells that dampen host immune responses, facilitating extracellular growth [3–6]. Members of human pathogenic *Yersinia* spp. include *Yersinia pestis*, the causative agent of plague, and the enteropathogens *Yersinia enterocolitica* and *Yersinia pseudotuberculosis*. While the T3SS is critical for infection, this apparatus appears to be metabolically burdensome since T3SS activity leads to growth arrest [7,8]. In addition, the Ysc T3SS is associated with pathogen-associated molecular patterns (PAMPs) recognized by several innate immune receptors, and some of these T3SS-associated PAMPS have evolved under selective evolutionary pressure by the ensuing immune response [5,9]. Without tight regulation of T3SS expression and deployment, these metabolic and immunological burdens would decrease the chance of *Yersinia* survival in the host.

The Ysc T3SS is encoded on a 70 kb plasmid for *Y**ersinia* Virulence, known as pYV or pCD1 [10]. Transcriptional regulation of T3SS genes is maintained by a master regulator called LcrF/VirF [11–14]. LcrF itself is also encoded on pYV, within the *yscW-lcrF* operon, and is highly conserved among all three human pathogenic *Yersinia* spp. LcrF is part of a larger family of AraC-like transcriptional regulators, and orthologs exist in other T3SS-encoding pathogens, such as ExsA in the nosocomial pathogen *Pseudomonas aeruginosa* [15]. The *yscW-lcrF* operon is regulated by various mechanisms in response to different environmental stimuli, including temperature, oxygen, and iron availability [16,17]. For example, an RNA thermometer blocks the ribosome binding site of *lcrF* at room temperature, but melts at mammalian body temperature, allowing *lcrF* translation [16].

Temperature-dependent transcriptional control of *yscW-lcrF* has been previously linked to the Histone-like Nucleoid structuring protein, H-NS, and an H-NS-binding protein called YmoA (*Y**ersinia* modulator) [16]. H-NS contains an N-terminal oligomerization domain and a C-terminal DNA minor-groove binding domain separated by a flexible linker [18,19]. H-NS preferentially binds AT rich regions of DNA [19,20]. Once H-NS binds a high-affinity site, H-NS oligomerizes on the DNA [21,22]. H-NS oligomers can either form a nucleoprotein filament on a contiguous stretch of DNA, or H-NS can form DNA bridges when multiple discrete H-NS binding regions are brought together, either way leading to transcriptional silencing of that particular gene [23]. Interestingly, H-NS in multiple bacterial pathogens has been shown to silence certain gene targets during growth outside of the mammalian host (20–30°C), but fails to silence these targets to the same magnitude when exposed to mammalian body temperature (37°C) [24–26]. This suggests H-NS may play a role in repressing virulence factors outside host organisms in facultative pathogens. However, H-NS is an essential gene in pathogenic *Yersinia* [27–30], making it challenging to definitively test the role of H-NS in regulating gene expression in these organisms.

YmoA, an *E*. *coli* Hha (“high hemolysin activity”) ortholog, has been suggested to modulate H-NS repression of a subset of promoters and deletion of *ymoA* in *Yersinia* leads to changes in gene expression of putative H-NS targets [16,31–33]. YmoA and Hha lack a DNA binding domain; instead, these proteins form a heterocomplex with H-NS or H-NS paralogs [34–37]. Recent data has suggested that Hha contributes to H-NS silencing by aiding in H-NS bridging [38]. While YmoA alone cannot bind the *yscW-lcrF* promoter, H-NS alone or the H-NS/YmoA complex can [16]. In *Y*. *pestis*, YmoA is suggested to have a higher turnover rate at 37°C compared to environmental temperatures [32]. Current models suggest that degradation of YmoA, and therefore a reduction in the H-NS/YmoA complex at 37°C, relieves repression of *yscW-lcrF* [32]. Yet, *ymoA* deletion mutants exhibit higher levels of T3SS expression at 37°C compared to a parental strain in all three pathogenic *Yersinia* species [16,31,32], suggesting that some YmoA is present at 37°C during mammalian infection, and that H-NS retains repressive activity.

The Iron Sulfur Cluster Regulator IscR is a critical positive regulator of *lcrF* [17,39]. IscR belongs to the Rrf2 family of winged helix-turn-helix transcription factors [40,41]. IscR was first characterized in *E*. *coli* where it exists in two forms: holo-IscR bound to a [2Fe-2S] cluster, and cluster-less apo-IscR [42–45]. Both forms of IscR bind DNA, but while both apo-IscR and holo-IscR bind to so-called type II motif sequences, only holo-IscR binds type I motifs [43,44]. Holo-IscR represses its own expression through binding two type I motifs in the *isc* promoter [46]. Thus, conditions that increase iron-sulfur cluster demand, such as iron starvation or oxidative stress, lead to a lower holo- to apo-IscR ratio and higher overall IscR levels. We have previously shown that low iron and oxidative stress lead to upregulation of IscR in *Yersinia*, and subsequently upregulation of *lcrF* transcription and T3SS expression at 37°C [17,39].

In this study, we further analyze the role of H-NS and YmoA in regulation of the *yscW-lcrF* promoter at 37°C and integrate this into our current understanding of positive regulation by IscR. Promoter truncations and site mutations were used to probe the cis-acting sequences in the *yscW-lcrF* promoter region that are required for H-NS/YmoA repressive activity. Direct DNA binding of H-NS and IscR to this upstream region was investigated by ChIP-qPCR. A requirement for H-NS in controlling *lcrF* expression was examined using a CRISPRi mediated *hns* knockdown strain. The results revealed a specific role of H-NS in repression of *lcrF*, identified two H-NS binding regions in the *yscW-lcrF* promoter required for H-NS/YmoA repression, and showed that disruption of the H-NS/YmoA complex eliminates repression. Furthermore, our results reveal a surprising finding that the positive impact of IscR is eliminated in strains defective in formation of the H-NS/YmoA repressive complex. Taken together, our data suggest that an H-NS/YmoA complex is critical for proper IscR-dependent regulation of LcrF and the T3SS in response to changes in oxygen tension.

## Results

### Knockdown of H-NS leads to derepression of LcrF

**Fig 1:**
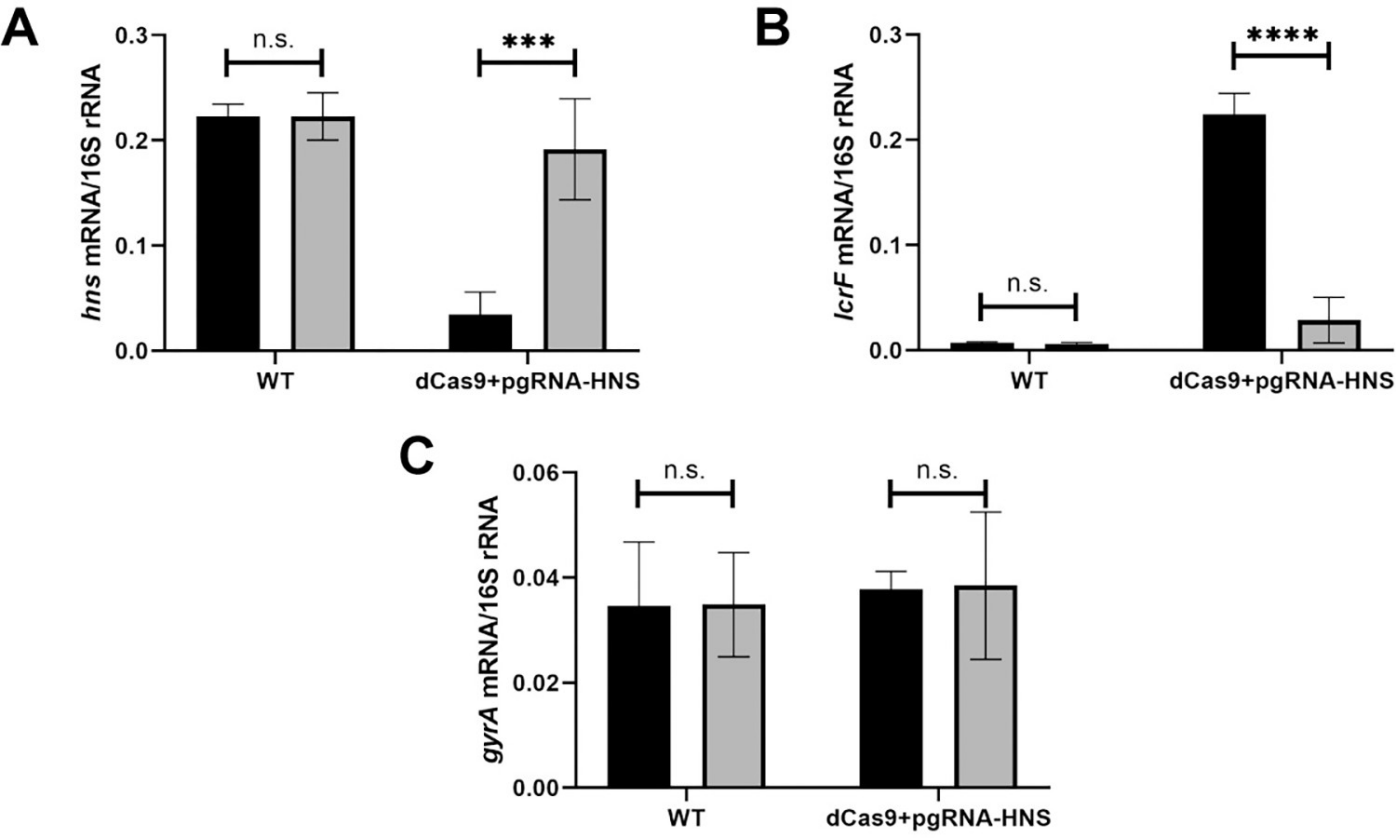
Knockdown of H-NS leads to derepression of LcrF. *Y*. *pseudotuberculosis* strains were grown aerobically in low calcium LB in the absence (grey bars) or presence (black bars) of anhydrotetracycline for 3 hrs at 26°C to induce expression of *hns* guide RNA and dCas9 and then transferred to 37°C (T3SS inducing conditions) for 1.5 hrs. RNA was analyzed by RT-qPCR for *hns* **(A)**, *lcrF* **(B)**, or *gyrA* **(C)** mRNA level normalized to 16S rRNA. The average of three biological replicates are shown ± standard deviation. Statistical analysis was performed using an unpaired Student’s t-test (***p < .001, ****p < .0001, and n.s. non-significant).

H-NS is essential in human pathogenic *Yersinia* [27–30], complicating analysis of its involvement in Ysc T3SS expression. Therefore, to test the involvement of H-NS in *lcrF* regulation, we used CRISPRi to knock down H-NS expression in *Y*. *pseudotuberculosis* and measured the effect on *lcrF* expression levels at 37°C. For this CRISPRi system pioneered in *Y*. *pestis* [47], target gene guide RNAs and dCas9 can be induced in the presence of anhydrotetracycline (aTC). CRISPRi knockdown led to a ~6-fold decrease in H-NS transcription when exposed to aTC (Fig 1A). Importantly, this reduction of H-NS expression led to a ~31-fold increase in *lcrF* mRNA, demonstrating that H-NS represses LcrF transcription (Fig 1B). Knockdown of H-NS did not affect expression of *gyrA*, a housekeeping gene not predicted to be regulated by H-NS (Fig 1C). These data provide the first direct evidence that H-NS negatively influences *Yersinia* LcrF expression at 37°C.

### H-NS occupies three DNA sites within the *yscW-lcrF* promoter

**Fig 2:**
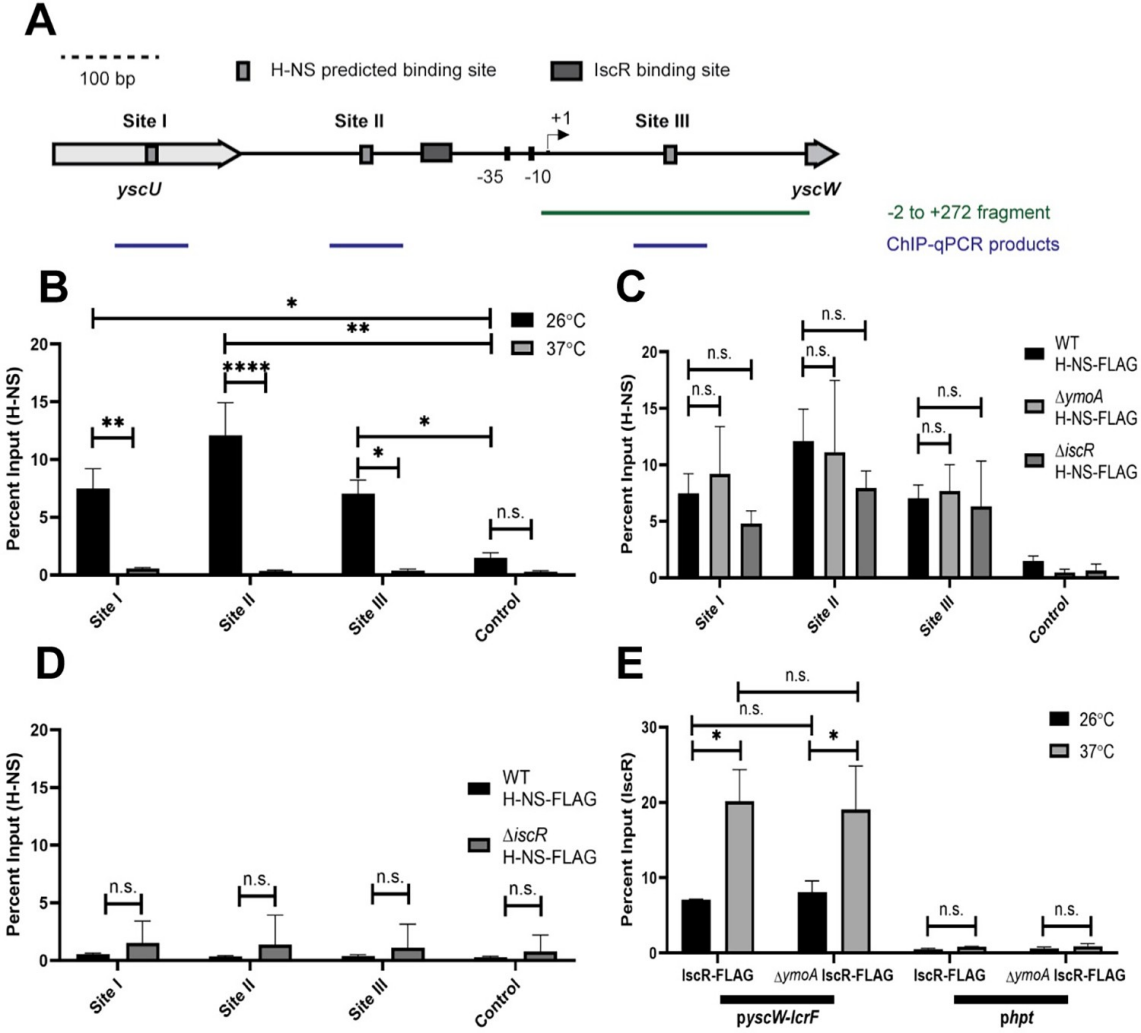
H-NS and IscR enrichment at the *yscW-lcrF* promoter. **(A)** FIMO-MEME suite tools were used to identify H-NS binding sites in the *yscW-lcrF* promoter. Shown are the three putative sites identified (p<10^−3^), referred to as Sites I, II, and III. The previously characterized transcriptional start site (TSS) is shown by the arrow [16], and the known IscR binding site is shown by the grey rectangle [39]. The -2 to +272 DNA fragment (relative to the +1 TSS) previously shown to bind H-NS *in vitro* [16] is shown, as well as the qPCR products detected following H-NS chromatin immunoprecipitation. **(B)** The relative enrichment (percent input) of Site I, Site II, and Site III promoter DNA and a negative control promoter (DN756_21750) was analyzed by anti-FLAG ChIP-qPCR in *Yersinia* expressing H-NS-FLAG. ChIP-qPCR was performed with bacteria grown aerobically at 26°C (black bars) or 37°C (grey bars) in low calcium LB for 3 hrs. **(C)** ChIP-qPCR was performed with the H-NS-FLAG allele in WT, Δ*ymoA*, or Δ*iscR* mutant background at 26°C **(D)** ChIP-qPCR was performed with the H-NS-FLAG allele in WT or Δ*iscR* mutant background at 37°C. **(E)** ChIP-qPCR was performed with the IscR-FLAG allele in the WT or Δ*ymoA* mutant background at 26°C (black bars) or 37°C (grey bars). The *hpt* control promoter, which IscR is not predicted to bind, was used as a negative control. The average of at least three biological replicates ± standard deviation is shown and statistical analysis was performed using Two-way ANOVA (*p < .05, **p < .01, ***p < .001, ****p < .0001 and n.s. non-significant).

H-NS and H-NS/YmoA complexes have been shown *in vitro* to bind a *yscW-lcrF* promoter fragment containing sequences between the -2 to +272 nucleotides relative to the transcriptional start site [16]. However, the specific H-NS binding regions were not identified. We used FIMO-MEME suite tools to predict putative H-NS binding sites upstream of *yscW-lcrF* and identified three predicted H-NS binding sites, referred to as Sites I, II, and III. Site III is contained within the -2 to +272 nucleotide sequence originally shown to interact with H-NS, and Site I and II are located further upstream (Fig 2A). To test whether H-NS binds to these predicted sites, we carried out ChIP-qPCR analysis to assess H-NS occupancy *in vivo*. In order to immunoprecipitate H-NS-DNA complexes, we used a chromosomally-encoded 3xFLAG tagged H-NS allele. This FLAG tag did not affect the ability of H-NS to repress LcrF expression (Fig A in S1 Text). Consistent with this, H-NS enrichment was readily detectable at all three predicted sites in the *yscW-lcrF* promoter when bacteria were cultured at 26°C. However, H-NS binding at these predicted sites was greatly diminished at 37°C (Fig 2B), despite the fact that H-NS was required for repression of *yscW*-*lcrF* at this temperature. Interestingly, previous reports have shown that H-NS in other facultative pathogens represses expression of certain virulence genes at environmental temperatures (<30°C) but exhibits decreased binding at mammalian body temperature (37°C) [24,26,48], in line with our findings. No enrichment of H-NS was seen at a control pYV-encoded promoter that was not predicted to bind H-NS at either temperature (DN756_21750). These data suggest that H-NS occupies the *yscW-lcrF* promoter at high levels under environmental temperatures at which the T3SS is known to be efficiently repressed [11,16]. Nevertheless, it is likely that some H-NS binds to the *yscW-lcrF* promoter at 37°C, but is below the limit of detection of ChIP-qPCR.

YmoA is predicted to enhance the repressive ability of H-NS on the *yscW-lcrF* promoter at both 25°C and 37°C, but previous *in vitro* data suggests that this effect is not mediated by increasing H-NS binding to the *yscW-lcrF* promoter [16]. Consistent with this, no difference in H-NS enrichment at the *yscW-lcrF* promoter was observed *in vivo* by ChIP-qPCR in the *ymoA* mutant compared to the parental strain at 26°C, suggesting that YmoA does not affect H-NS occupancy at the *yscW-lcrF* promoter at this temperature (Fig 2C). In addition, deletion of *ymoA* did not change our ability to detect H-NS binding to the *yscW-lcrF* promoter at 37°C. Since previous data showed that IscR exerts its positive effect at 37°C, we also assayed H-NS enrichment in a strain lacking IscR and found no difference between an *iscR* mutant and the wildtype strain at 26°C (Fig 2C) or 37°C (Fig 2D). Taken together, these data indicate that H-NS binds to the three predicted DNA sites in the *yscW-lcrF* promoter *in vivo* and occupancy of these sites is higher at environmental temperatures compared to 37°C but is independent of IscR or YmoA.

### Two H-NS binding regions are required to repress *yscW-lcrF* promoter activity

**Fig 3:**
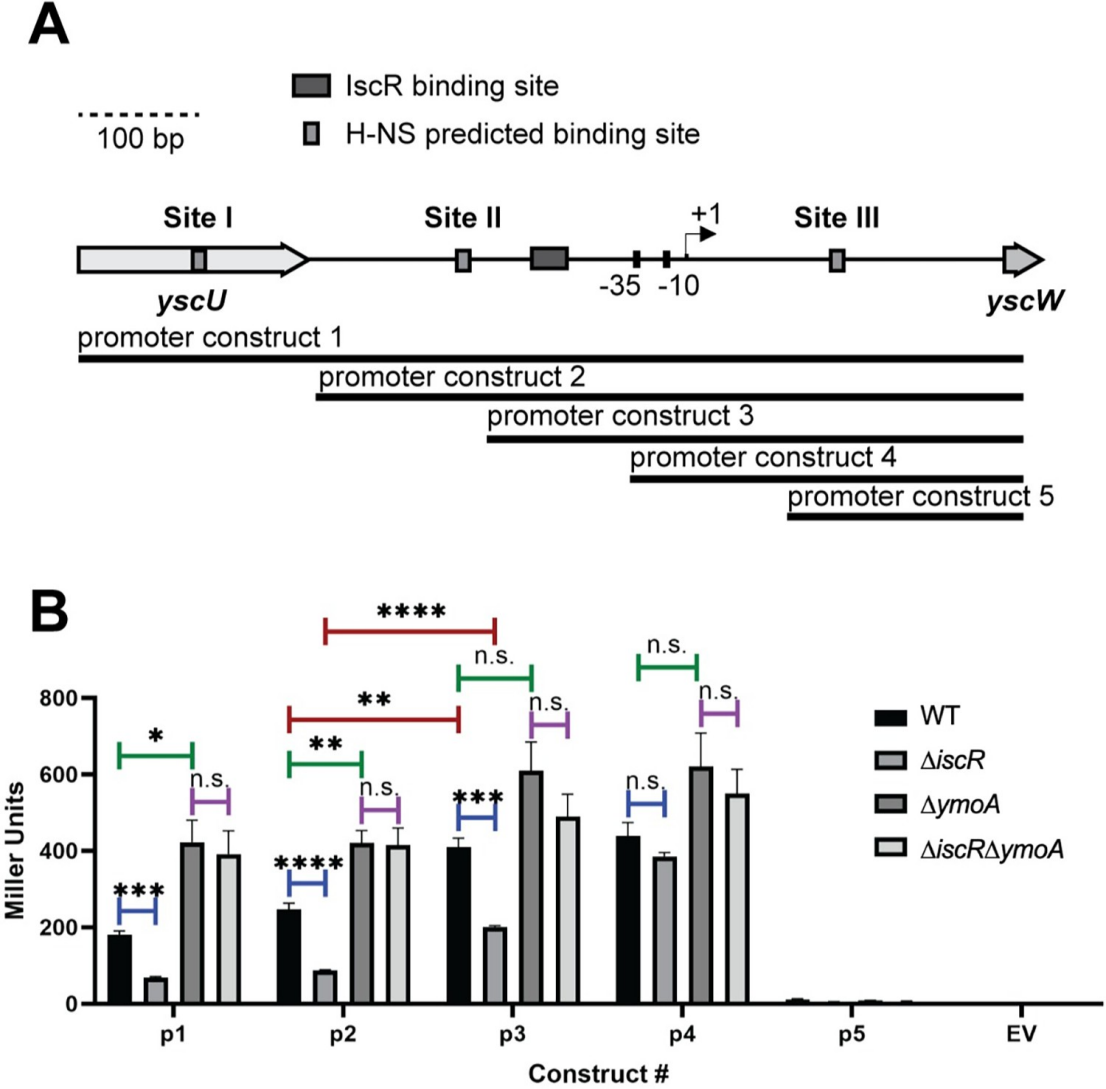
The identified H-NS binding Sites II and III are important for regulation of *yscW-lcrF* promoter activity. **(A)** Schematic of P*yscW-lcrF*::*lacZ* fusions. Five constructs (p1-p5) were used to assess which regions of p*yscW-lcrF* allows for H-NS-YmoA repression and IscR activation. **(B)** *Yersinia* harboring the various p*yscW-lcrF*::*lacZ* plasmids were grown aerobically under T3SS-inducing conditions (low calcium LB at 37°C) for 1.5 hrs and assayed for β-galactosidase (Miller units). The average of at least three biological replicates are shown ± standard deviation. Statistical analysis was performed using an unpaired Student’s t-test (*p < .05, **p < .01, ***p < .001, ****p < .0001, and n.s. non-significant).

To determine whether any of the three identified H-NS binding Sites I, II, and III function in H-NS-dependent regulation of the *yscW-lcrF* promoter at 37°C, we systematically truncated the *yscW-lcrF* promoter and tested promoter activity using a *lacZ* reporter (Fig 3A). We also compared expression in a mutant lacking YmoA as a close mimic for strains lacking H-NS since *ymoA* is not an essential gene, whereas *hns* cannot be deleted. Eliminating the region containing H-NS binding Site I did not significantly affect promoter activity (Fig 3B; promoter 1 compared to promoter 2). However, additional truncation of H-NS binding Site II led to a significant increase in promoter activity that was less sensitive to YmoA, suggesting that some of the H-NS repressive effect had been lost by Site II truncation (Fig 3B; promoter 2 compared to promoter 3). Importantly, further truncation to eliminate the IscR binding site led to deregulated promoter activity (Fig 3B; promoter 4). Lastly, truncation to eliminate the -35 and -10 promoter elements led to a complete lack of promoter activity (Fig 3B; promoter 5), as expected. Taken together, these data suggest that H-NS binding Sites II and III are required for repression of *yscW-lcrF* promoter activity.

**Fig 4:**
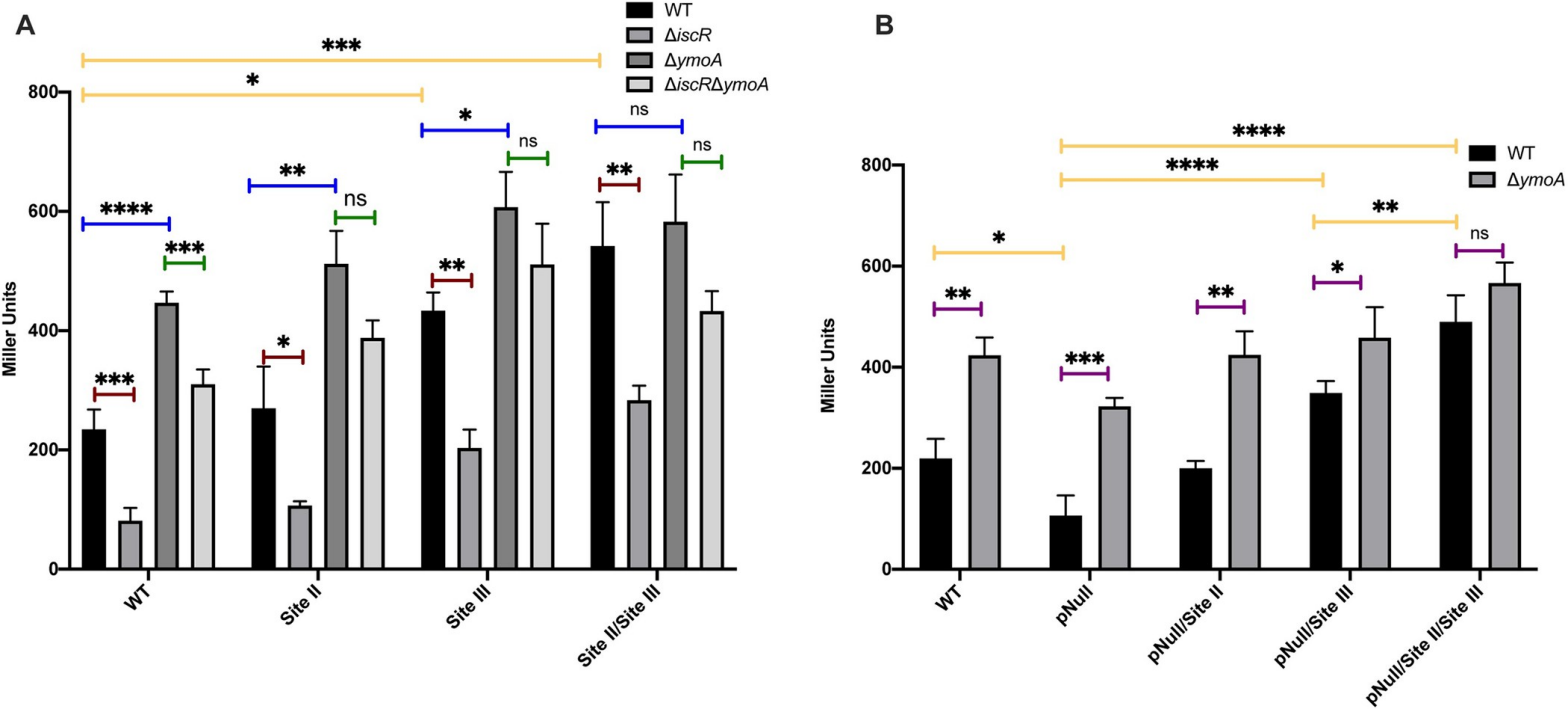
The AT-rich Sites II and III bound by H-NS are necessary for YmoA-dependent repression as well as IscR potentiation of *yscW-lcrF* promoter activity. **(A)** The *pyscW-lcrF*::*lacZ* reporter 2 construct from Fig 3 was used as a template to generate *pyscW-lcrF*::*lacZ* promoter fusions with H-NS Site II and III mutations, and the mutated promoters introduced into the WT, Δ*iscR*, Δ*ymoA*, and Δ*iscR*/Δ*ymoA Y*. *pseudotuberculosis* genetic backgrounds. **(B)** Additionally, *pyscW-lcrF*::*lacZ* reporter 2 constructs carrying WT or mutated Site II and/or III were further mutated for the IscR binding site (pNull) and introduced into WT and Δ*ymoA Y*. *pseudotuberculosis*. All these strains were grown aerobically under T3SS-inducing conditions (low calcium LB at 37°C) for 1.5 hrs and assayed for β-galactosidase (Miller units). The different promoter constructs are listed on the x-axis. The average of at least three biological replicates are shown ± standard deviation. Statistical analysis was performed using **(A-B)** a one-way ANOVA with Bonferroni’s multiple comparisons test on either (yellow bars) all WT genetic backgrounds carrying different reporter constructs or (blue, green, or red bars) each individual reporter construct expressed in WT, Δ*iscR*, Δ*ymoA*, or Δ*iscR*/Δ*ymoA*; or **(B)** an unpaired t test (purple bars). (*p < .05, **p < .01, ***p < .001, ****p < .0001, and n.s. non-significant).

Since Site III is downstream of the transcription initiation site, we could not assess its contribution through promoter truncation. Thus, we mutated Site III in the *yscW-lcrF* promoter 2 *lacZ* construct by switching the TA-rich sequences to CG-rich sequences to perturb H-NS binding and compared it to a similarly mutated Site II. Mutation of Site III resulted in significantly increased promoter activity in the WT genetic background (Fig 4A). Mutation of Site II did not lead to a statistically significant increase (Fig 4A, WT background), although mutation of both Site II and Site III resulted in promoter activity that trended higher than mutation of Site III alone. Importantly, mutation of Site II and/or III did not significantly change promoter activity in the absence of YmoA (Fig 4A, Δ*ymoA* background), suggesting YmoA mediated repression was eliminated. Thus, these data suggest that H-NS binding to at least two of the three identified binding regions plays a major role in H-NS-mediated repression of the *yscW-lcrF* promoter.

Given the location of the IscR binding site between H-NS Sites II and III, we also determined whether IscR regulation of the *yscW-lcrF* promoter is impacted by H-NS/YmoA-mediated repression. While deletion of *iscR* did not affect YmoA or H-NS expression (Fig B in S1 Text), we found that deletion of *iscR* led to a decrease in *yscW-lcrF* promoter activity compared to the wildtype strain for all promoter constructs containing the known IscR binding site (Fig 3; promoters 1–3), as expected from our previous studies [17,39]. Surprisingly, in the absence of *ymoA*, deletion of *iscR* had no effect on promoter activity (Fig 3B), suggesting that IscR is dispensable for *yscW-lcrF* promoter activity in the absence of an H-NS/YmoA-repressive complex.

Since IscR had no effect on *yscW-lcrF* promoter activity in the absence of YmoA, we tested whether IscR enrichment at the *yscW-lcrF* promoter *in vivo* was impacted by YmoA. We used a chromosomal 3xFLAG tagged IscR allele previously shown not to affect IscR activity [49]. IscR enrichment at the *yscW-lcrF* promoter was not influenced by the presence of YmoA since the levels were the same between the wildtype strain and the *ymoA* mutant at either 26° or 37°C (Fig 2E). However, IscR enrichment at the *yscW-lcrF* promoter was ~3-fold higher at 37°C compared to 26°C, while IscR enrichment at the promoter of another known IscR target, the *suf* operon, did not significantly differ with temperature (Fig C in S1 Text). This increase in IscR enrichment at the *yscW-lcrF* promoter at 37°C is not due to increased IscR levels since we did not observe higher levels of IscR protein when *Yersini*a was cultured at 37°C compared to 26°C (Fig B in S1 Text). Thus, a lack of IscR binding cannot explain the absence of IscR regulation of the *yscW-lcrF* promoter in strains deleted for *ymoA*. Rather, based on the results establishing a repressive effect of an H-NS/YmoA complex acting through H-NS binding to regions flanking the IscR binding site, we considered the notion that IscR acts by interfering with H-NS/YmoA repression of the *yscW-lcrF* promoter.

To test whether IscR potentiation of *yscW-lcrF* promoter activity was affected by the H-NS/YmoA complex, we mutated the characterized IscR binding site in the *yscW-lcrF* promoter 2 *lacZ* construct (*yscW-lcrF*^pNull^). We previously showed that this mutation ablated IscR binding [17]. As expected, a reporter lacking just the IscR binding site had two-fold less promoter activity than the wildtype construct (Fig 4B, WT background). Interestingly, mutating both Site II and III in the *yscW-lcrF*^pNull^ construct led to significantly higher promoter activity than mutating only Site III, supporting the assertion stated above that Site II contributes to *yscW-lcrF* repression (Fig 4B, WT background). In contrast, mutation of Site II and III in the *yscW-lcrF*^pNull^ construct did not lead to significant change in promoter activity in the absence of YmoA (Fig 4B, Δ*ymoA* background), suggesting that the repressive effect of Sites II and III requires YmoA. Collectively, these data show that while both IscR binding to the *yscW-lcrF* promoter and the H-NS binding Sites II and III regulate *yscW-lcrF* promoter activity in opposing directions, IscR is dispensable for *yscW-lcrF* promoter activity in the absence of H-NS/YmoA-mediated repression.

### IscR is not required for LcrF expression or type III secretion in the absence of YmoA

**Fig 5:**
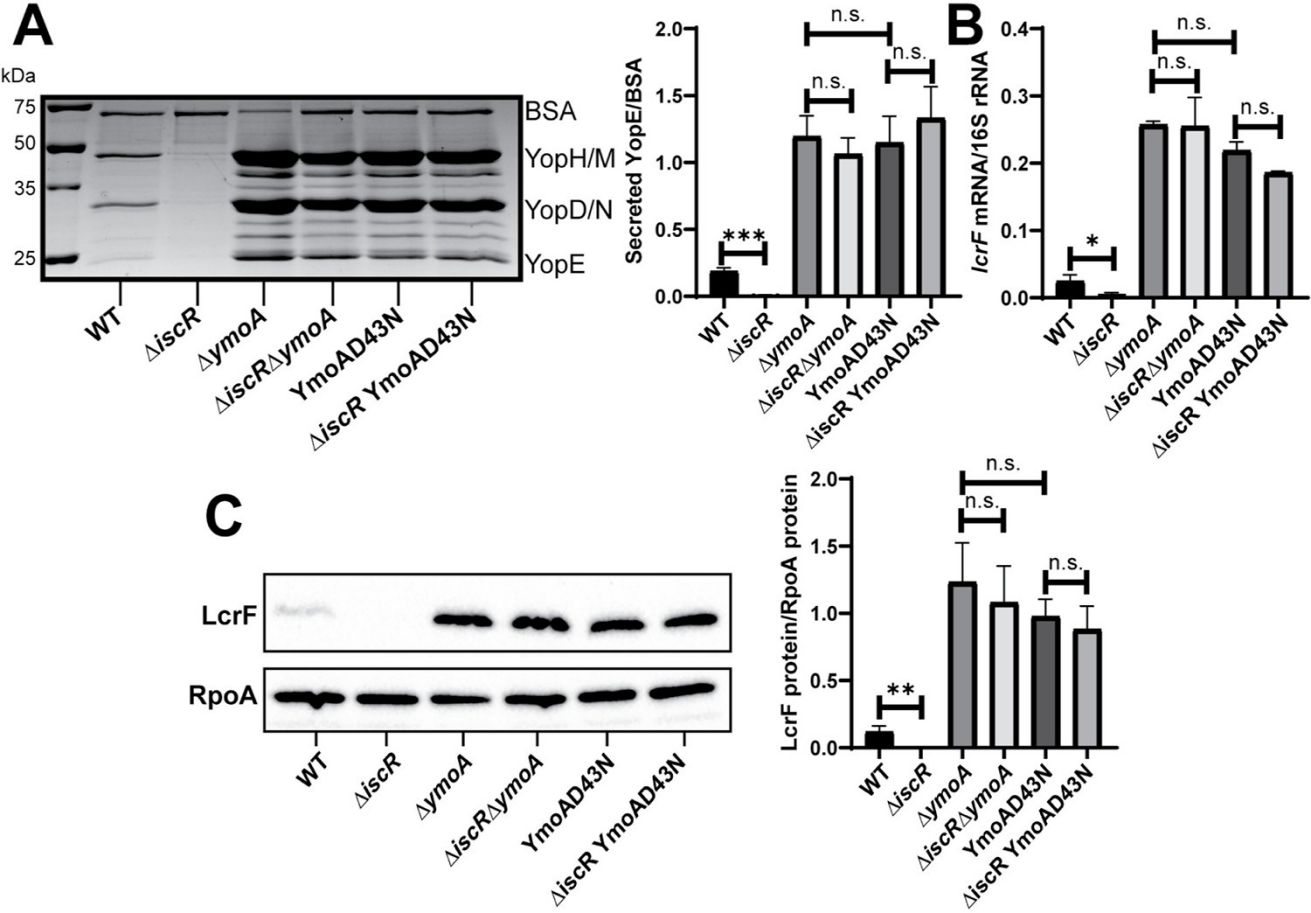
IscR is dispensable for type III secretion in the Δ*ymoA* mutant background. *Yersinia* strains were grown aerobically under T3SS-inducing conditions (low calcium at 37°C). **(A)** Precipitated secreted proteins were visualized by SDS-PAGE followed by Coomassie blue staining. Bovine serum albumin (BSA) was used as a loading control (left panel). Densitometry was used to measure the relative amount of secreted YopE T3SS effector protein versus BSA control. The average of four independent replicates ± standard deviation is shown (right panel). **(B)** RNA was extracted and RT-qPCR was used to measure relative levels of *lcrF* mRNA normalized to 16S rRNA. The average of at least three biological replicates are shown ± standard deviation. **(C)** LcrF protein levels were determined by Western blotting (left panel) and densitometry (right panel) relative to the RpoA loading control. Shown is the average of four independent replicates ± standard deviation. Statistical analysis was performed using an unpaired Student’s t-test (*p < .05, **p < .01, ***p < .001, and n.s. non-significant).

Since we found that loss of *iscR* did not decrease *yscW-lcrF* promoter activity in the absence of YmoA, we assessed T3SS activity of *Y*. *pseudotuberculosis* expressing or lacking *iscR* and/or *ymoA*. Consistent with previous studies, we observed ~18-fold decrease in secretion of the T3SS effector protein YopE upon *iscR* deletion, while *ymoA* deletion led to ~6-fold increase in YopE secretion (Fig 5A). As expected, the effect of YmoA on T3SS activity required LcrF (Fig D in S1 Text). Importantly, YopE secretion in the Δ*iscR*/Δ*ymoA* double mutant was similar to the Δ*ymoA* mutant, indicating that IscR is dispensable for T3SS activity in the absence of YmoA (Fig 5A). As proteins of the YmoA family lack a DNA binding domain and are thought to affect transcription by interacting with H-NS [33,38], we tested whether the ability of YmoA to bind H-NS was required for its ability to control *lcrF* expression and, therefore, T3SS activity. Previous studies showed that a YmoA D43N mutant cannot interact with H-NS *in vitro* [50]. A *Y*. *pseudotuberculosis ymoA*^D43N^ mutant was able to express YmoA (Fig E in S1 Text), but exhibited ~6-fold increase in YopE secretion, similar to *ymoA* deletion (Fig 5A). This suggests that YmoA represses *yscW-lcrF* through its interaction with H-NS. Furthermore, there was no difference in YopE secretion between the *ymoA*^*D43N*^ mutant and the *iscR*/*ymoA*^*D43N*^ double mutant. These effects on YopE secretion are most easily explained by changes in LcrF levels. As expected, the Δ*iscR* mutant had a ~5-fold reduction in *lcrF* mRNA compared to wildtype, while the Δ*ymoA* and *ymoA*^D43N^ mutants displayed ~10-fold elevated *lcrF* mRNA (Fig 5B and 5C). In contrast, we observed no difference in LcrF expression between the Δ*ymoA* and Δ*iscR/ΔymoA* mutants or between the *ymoA*^D43N^ and Δ*iscR*/*ymoA*^D43N^ mutants. Likewise, mutation of the IscR binding site mutant in the *yscW-lcrF* promoter (*lcrF*^pNull^) reduced LcrF expression and T3SS activity in the presence of YmoA, but not in the absence of YmoA (Fig F in S1 Text). Collectively, these data suggest that YmoA requires H-NS binding to inhibit *lcrF* transcription, and that IscR regulation of the *yscW*-*lcrF* promoter and T3SS expression is only important in the presence of the H-NS/YmoA complex.

### IscR-dependent regulation of LcrF and the T3SS in response to oxygen availability requires YmoA

**Fig 6:**
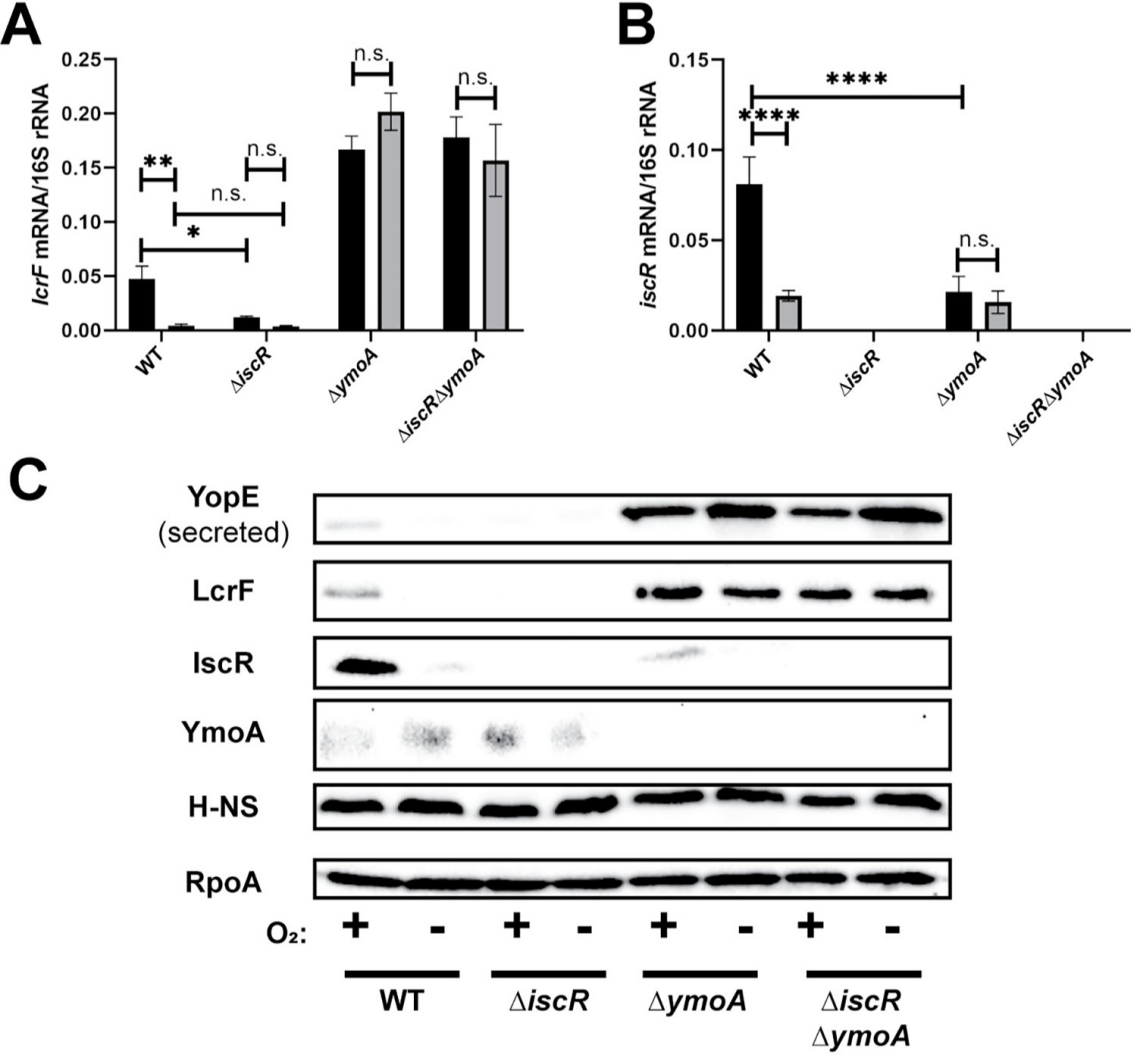
Oxygen-dependent control of *lcrF* requires YmoA. *Yersinia* strains were cultured under T3SS inducing conditions under aerobic (black bars) or anaerobic (grey bars) conditions. Levels of *lcrF* **(A)** and *iscR* **(B)** mRNA levels were measured by RT-qPCR and normalized to 16S rRNA. The average of at three biological replicates are shown ± standard deviation. **(C)** *Yersinia* strains were grown under similar conditions as stated above and whole cell extracts were probed for RpoA, IscR, H-NS, LcrF, YopE, and YmoA by Western blotting. One representative experiment out of three biological replicates is shown. Statistical analysis was performed using a one-way ANOVA with Tukey multiple comparisons (*p < .05,**p < .01,****p < .0001, and n.s. non-significant).

We previously showed that low iron and high oxidative stress lead to elevated IscR levels, which then activate T3SS expression through upregulation of LcrF [17]. The data shown here suggest that IscR is dispensable in the absence of H-NS/YmoA activity at the *yscW-lcrF* promoter. To test how YmoA affects the ability of IscR to regulate LcrF in response to environmental cues, we measured *lcrF* mRNA levels in Δ*iscR* and Δ*ymoA* mutants under aerobic or anaerobic conditions at 37°C. As expected, under aerobic conditions *iscR* mRNA levels were increased ~4-fold compared to anaerobic conditions, and this upregulation of *iscR* levels led to a ~12-fold induction in *lcrF* levels in the wildtype strain (Fig 6A and 6B). In contrast, *lcrF* mRNA and protein levels were not affected by oxygen in the Δ*ymoA* and Δ*iscR/ΔymoA* mutants (Fig 6A and 6C). Unlike what we observed using rich media (Fig B in S1 Text), deletion of *ymoA* reduced expression of IscR mRNA and protein expression under these minimal media conditions (Fig 6B and 6C), although this does not explain elevated LcrF/T3SS expression in the *ymoA* mutant. Taken together, these data suggest that the H-NS/YmoA complex facilitates IscR regulation of LcrF and the T3SS in response to environmental cues.

## Discussion

The histone-like protein H-NS has been suggested to repress expression of horizontally transferred genes, but the *hns* gene is essential in pathogenic *Yersinia* [27–30]. In this study, CRISPRi knockdown in *Y*. *pseudotuberculosis* revealed that *hns* is required for repression of the LcrF T3SS master regulator. Furthermore, genetic analysis revealed that YmoA must interact with H-NS to repress *lcrF* transcription. We identified two relevant H-NS binding sites in the *yscW-lcrF* promoter that contribute to promoter repression in a manner dependent on YmoA. Interestingly, we found that the transcription factor IscR promotes *lcrF* expression and T3SS activity only in the presence of H-NS/YmoA. *Y*. *pseudotuberculosis* IscR levels are low under anaerobic conditions, such as those in the intestinal lumen. As *Yersinia* cross the intestinal barrier, oxygen tension increases. Oxidative stress has been shown to stimulate elevated IscR levels that drive LcrF expression and type III secretion, which is critical for extraintestinal infection [17,51–54]. Importantly, oxygen- and IscR-dependent regulation of LcrF and the T3SS required an H-NS/YmoA complex. These data suggest that following acquisition of the pYV virulence plasmid encoding the Ysc T3SS during *Yersinia* evolution, Ysc T3SS genes may have come under general repression by H-NS and its modulator YmoA but evolved to respond specifically to IscR-responsive host tissue cues to enable T3SS-mediated target host cell immunomodulation.

YmoA was previously shown to bind H-NS and the H-NS/YmoA complex was proposed to regulate LcrF expression [16,50]. Indeed, a YmoA point mutation that eliminates H-NS binding phenocopied the *ymoA* deficient strain, suggesting YmoA must interact with H-NS to repress LcrF and T3SS expression. However, YmoA was not shown to affect H-NS binding to the *yscW-lcrF* promoter *in vitro* [16], and our ChIP-qPCR analysis did not find a change in H-NS *yscW-lcrF* promoter occupancy in the presence or absence of YmoA at 26°C. At 37°C, H-NS occupancy was below the limit of detection by ChIP-qPCR, so we could not rule out H-NS binding to the *yscW-lcrF* promoter at this temperature, nor the effect of YmoA on residual H-NS binding. However, deletion of *ymoA* or knockdown of *hns* both caused elevated LcrF expression at 37°C, indicating that both proteins are needed to repress the *yscW-lcrF* promoter at mammalian body temperature. Furthermore, the fact that mutation of the Site II/III H-NS binding sites in an *lcrF* transcriptional reporter construct led to an increase in promoter activity at 37°C suggests that H-NS repression of *lcrF* activity is at least partially due to direct binding. However, it is possible that H-NS may have additional indirect effects on *lcrF* transcription. A recent study showed that deletion of *ymoA* impacts expression of almost 300 genes, including known regulators [55], raising the possibility that deletion of *ymoA* may affect the *yscW-lcrF* promoter both directly and indirectly. However, the fact that deletion of YmoA did not affect promoter activity of the *yscW-lcrF* Site II/III mutant promoter construct suggests that YmoA does not affect *yscW-lcrF* promoter activity independently of the H-NS binding sites. Taken together, these data suggest that YmoA binding to H-NS does not alter H-NS occupancy at the *lcrF* promoter but, rather, potentiates H-NS repressive activity. How YmoA affects H-NS activity will need to be addressed by future studies. Importantly, a *Y*. *pseudotuberculosis* strain lacking *ymoA* is attenuated in colonization of intestinal lymph tissue and vital organs following oral infection [55]. While derepression of the T3SS may play a role in this virulence defect, the effect of *ymoA* deletion on expression of *Yersinia* adhesins and other virulence factors likely contributes [55]. Indeed, YmoA modulates the carbon storage regulator (Csr) global regulatory system known to regulate several virulence factors. We observed that *ymoA* deletion led to lower IscR levels following growth in minimal media (Fig 6) but not in rich media (Fig B in S1 Text), suggesting that perhaps YmoA contributes to *iscR* expression under nutrient limiting conditions.

The pYV plasmid in the region of the *lcrF* gene has been reported to take on different architecture depending on temperature [56]. A “bent” pYV architecture was suggested to be stabilized by H-NS at lower temperatures while this bending was not detected at 37°C. H-NS oligomers can either form a nucleoprotein filament on a contiguous stretch of DNA or can form DNA bridges when multiple discrete H-NS binding regions are brought together [57,58]. It is possible that at lower temperatures, H-NS forms nucleoprotein filaments at the *lcrF* gene locus on pYV, perhaps nucleated from the identified binding sites, and thus H-NS enrichment via ChIP-qPCR is readily detectable. In contrast, it is possible that at 37°C, fewer H-NS molecules are bound and the sensitivity of ChIP-qPCR is not sufficient to detect this binding. However, the fact that mutation of the H-NS binding Sites II and III in the *yscW-lcrF* promoter leads to derepression of promoter activity at 37°C, and that this is independent of YmoA, strongly suggest that H-NS/YmoA is still active at the wildtype *yscW-lcrF* promoter at 37°C.

Previous reports have suggested that H-NS represses some target genes under environmental conditions, but to a lesser extent at mammalian body temperature. For example, the *Shigella flexneri* T3SS is co-regulated by the AraC transcriptional regulator VirF and H-NS [59,60]. VirF promotes VirB, which ultimately activates the *Shigella* T3SS [61]. The *Shigella* T3SS is only expressed at mammalian body temperature and this is controlled by preventing expression of VirF at environmental temperatures. H-NS binds to two distinct sites upstream of *virF* leading to the formation of a DNA bridge [62], repressing *virF* transcription [48]. Furthermore, more H-NS binding to the *virF* promoter is observed at lower temperatures (<30°C) compared to mammalian body temperature (37°C). Similarly, we observed H-NS enrichment at the *yscW-lcrF* promoter robustly at environmental temperature while it was below the limit of detection by ChIP-qPCR at 37°C. However, *Yersinia* H-NS repression of LcrF still plays an important role at 37°C, as CRISPRi knockdown at this temperature leads to *lcrF* derepression.

IscR-regulated genes in other bacterial pathogens have also been shown to be repressed by H-NS, and H-NS has been shown to repress T3SS genes in many bacterial species. In *Vibrio* species, for example, nitrosative stress and iron starvation drive *V*. *vulnificus* IscR to compete with H-NS binding to the promoter of the *vvhBA* operon, which encodes an extracellular pore-forming toxin essential for its hemolytic activity [63–66]. Furthermore, in *V*. *parahaemolyticus*, H-NS represses expression of the T3SS-1 regulator ExsA, while *exsA* is induced by the transcription factor HlyU [67]. Like IscR, HlyU may also sense oxygen levels [68]. In *Salmonella enterica*, H-NS has been suggested to bind to a high-affinity nucleation site in the regulatory region of the *Salmonella* pathogenicity island (SPI)-1 T3SS regulator HilD, leading to repression of *hilD* [69]. In addition, genetic evidence suggests that this H-NS repression of *hilD* can be counteracted by HilD itself [69]. Interestingly, IscR has been shown to repress the *Salmonella* pathogenicity island (SPI)-1 T3SS regulator HilD in response to iron depletion, possibly facilitating expression of the SPI-1 T3SS in the intestinal lumen where it is needed to enter intestinal epithelial cells [70]. Likewise, in *Pseudomonas aeruginosa*, the H-NS family members MvaT and MvaU repress the promoter of the T3SS master regulator ExsA, an AraC family transcription factor with similarity to *Yersinia* LcrF [11,71], while the cAMP-responsive Vfr protein directly activates *exsA* transcription [72]. Collectively, these data are consistent with the idea that T3SS genes must come under H-NS family repression following horizontal gene transfer, necessitating a positive regulatory factor that must overcome H-NS repression to induce virulence gene expression in response to an appropriate signal. In the case of the *Yersinia*, H-NS repression of the T3SS master regulator LcrF plays an active role in allowing T3SS genes to be responsive to relevant host tissue cues through IscR.

## Materials and methods

### Bacterial strains and growth conditions

Bacterial strains used in this paper are listed in Table A in S1 Text. *Y*. *pseudotuberculosis* were grown, unless otherwise specified, in LB (Luria Broth) at 26°C shaking overnight. To induce the T3SS, overnight cultures were diluted into low calcium LB medium (LB plus 20 mM sodium oxalate and 20 mM MgCl_2_) to an optical density (OD_600_) of 0.2 and grown for 1.5 h at 26°C shaking followed by 1.5 h at 37°C to induce Yop synthesis, as previously described [73].

For growing *Yersinia* under varying oxygen conditions, casamino acid-supplemented M9 media, referred to as M9 minimal media in this study, was used [74]. Growth of cultures to vary oxygen tension was achieved by first diluting 26°C overnight aerobic cultures of *Y*. *pseudotuberculosis* to an OD_600_ of 0.1 in fresh M9 minimal media supplemented with 0.9% glucose to maximize growth rate and energy production under anaerobic conditions, and incubating for 12 hrs under either aerobic or anaerobic conditions at 26°C. Both aerobic and anaerobic cultures were then diluted to an OD_600_ of 0.1, grown for 2 hrs at 26°C, and shifted to 37°C for 4 hrs.

### Construction of *Yersinia* mutant strains

The *Yersinia* mutants were generated as described in [49]. H-NS was tagged with a C-terminal 3xFLAG affinity tag at the native locus through splicing by overlap extension [75], using primer pair F*hns*_cds/R*hns*_cds (Table B in S1 Text) to amplify ~500bp upstream of *hns* plus the *hns* coding region excluding the stop codon, F3xFLAG/R3xFLAG to amplify the 3xFLAG tag, and F3’*hns*/R3’*hns* to amplify the ~500 bp downstream region of *hns* including the stop codon. For the Δ*ymoA* mutant, primer pairs F5/R5Δ*ymoA* were used to amplify ~1000 bp 5’ of *ymoA* and F3/R3Δ*ymoA* to amplify ~1000 bp 3’ of *ymoA*. To generate the *ymoA*^D43N^ mutant, primer pairs pUC19_YmoA_F and pUC19_YmoA_R were used to amplify 250 bp upstream of *ymoA* to 250 downstream of the *ymoA* start codon and the amplified product cloned into a BamHI and SacI digested pUC19 plasmid. Q5 site directed mutagenesis was performed using primer pairs *ymoA*^D43N^ _F and *ymoA*^D43N^ _R. The resulting plasmid, pUC19 *ymoA*^D43N^, was digested with BamHI and SacI and the resulting fragment was ligated into the suicide plasmid pSR47s. Mutant strains were generated as described above.

In order to generate *lacZ* promoter constructs of *ymoBA* and *hns*, primer pairs pFU99a_ymoA_F/pFU99a_ymoA_R and pFU99a_hns_F/pFU99a_hns_R were used to amplify ~500 bp upstream of *ymoA* and *hns*, respectively, which included the first ten amino acids of *ymoA* and *hns*. These promoters and first ten amino acids of YmoA and H-NS were fused in frame to *lacZ* and cloned into a BamHI- and SalI-digested pFU99a using the NEBuilder HiFi DNA Assembly kit (New England Biolabs, Inc) and electroporated into *Y*. *pseudotuberculosis*.

In order to generate *lacZ* promoter constructs of *yscW-lcrF*, the reverse primer pFU99a_*yscWlcrF*_R was used with the following forward primers: pFU99a_*yscWlcrF*_p1 (promoter construct 1/-505 to +294 of *yscW*), pFU99a_*yscWlcrF*_p2 (promoter construct 2/ -309 to +294 of *yscW*), pFU99a_*yscWlcrF*_p3 (promoter construct 3/ -166 to +294 of *yscW*), pFU99a_*yscWlcrF*_p4 (promoter construct 4/ -47 to +294 of *yscW*), or pFU99a_*yscWlcrF*_p5 (promoter construct 5/ +101 to +294 of *yscW*). These promoter fragments were cloned into a BamHI- and SalI-digested pFU99a and electroporated into *Y*. *pseudotuberculosis*. To generate the Site 2, Site 3, Site 2/Site3, pNull, pNull/Site2, pNull/Site3, and pNull/Site2/Site3 mutant *lacZ* promoter constructs, Q5 mutagenesis was used with primers listed in Table B in S1 Text. The single mutant plasmids were generated first, which were then used as templates to generate the double mutant plasmids, and the double mutant plasmid was used as a template to generate the triple mutant plasmid.

### Type III secretion system secretion assay

Visualization of T3SS cargo secreted in broth culture was performed as previously described [76]. Briefly, *Y*. *pseudotuberculosis* in LB low calcium media (LB plus 20 mM sodium oxalate and 20 mM MgCl2) was grown for 1.5 h at 26°C followed by growth at 37°C for 1.5 h. Alternatively for the ±O_2_ cultures, *Y*. *pseudotuberculosis* was grown in M9 minimal media with 0.9% glucose as described above. Cultures were normalized to OD_600_ and pelleted at 13,200 rpm for 10 min at room temperature. Supernatants were removed, BSA spiked into the supernatant to serve as a control, and proteins precipitated by addition of trichloroacetic acid (TCA) at a final concentration of 10%. Samples were incubated on ice for at least 1 hr and pelleted at 13,200 rpm for 15 min at 4°C. Resulting pellets were washed twice with ice-cold 100% acetone and resuspended in final sample buffer (FSB) containing 0.2 M dithiothreitol (DTT). Samples were boiled for 5 min prior to separating on a 12.5% SDS-PAGE gel. Coomassie stained gels were imaged using Bio-Rad Image Lab Software Quantity and Analysis tools. YopE bands were quantified using this software and normalized to the BSA protein precipitation control.

### Western blot analysis

Cell pellets were collected, resuspended in FSB plus 0.2 M DTT, and boiled for fifteen minutes. At the time of loading, supernatants and cell pellets were normalized to the same number of cells. After separation on a 12.5% SDS-PAGE gel, proteins were transferred onto a blotting membrane (Immobilon-P) with a wet mini trans-blot cell (Bio-Rad). Blots were blocked for an hour in Tris-buffered saline with Tween 20 and 5% skim milk, and probed with the rabbit anti-RpoA (gift from Melanie Marketon), rabbit anti-LcrF (gift from Gregory Plano), rabbit anti-IscR [44], rabbit anti-YmoA (gift from Gregory Plano), rabbit anti H-NS (gift from Robert Landick), mouse M2 anti-FLAG (Sigma), goat anti-YopE (Santa Cruz Biotech), and horseradish peroxidase-conjugated secondary antibodies (Santa Cruz Biotech). Following visualization, quantification of the bands was performed with Image Lab software (Bio-Rad).

### Quantitative RT-PCR

RT-qPCR was carried out as previously described [49] using the primers in Table B in S1 Text. The expression levels of each target gene were normalized to that of 16S rRNA present in each sample and calculated by utilization of a standard curve. At least three independent biological replicates were analyzed for each condition.

### β-galactosidase assays

*Y*. *pseudotuberculosis* harboring promoter-*lacZ* fusion plasmids were grown in LB low calcium media (LB plus 20 mM sodium oxalate and 20 mM MgCl2) for 1.5 h at 26°C followed by growth at 37°C for 1.5 h. Protein expression was stopped by incubating cells on ice for 20 minutes. Cultures were spun down and resuspended in Z Buffer [77]. Samples were permeabilized using chloroform and 0.1% sodium dodecyl sulfate, incubated with 0.8 mg/mL ONPG, and β-galactosidase enzymatic activity was terminated by the addition of 1M sodium bicarbonate. β-galactosidase activity is reported as Miller units.

### CRISPRi knockdown

Knockdown of H-NS via CRISPRi methods was adapted from [47]. In order to generate the pgRNA-tetO-JTetR-H-NS plasmid, a protospacer-adjacent motif (PAM) was located near the promoter of *hns* [78]. Two oligonucleotides (*hns*_gRNA_F and *hns*_gRNA_R) consisting of 20-nt targeting the *hns* promoter region with BbsI cohesive ends were synthesized and annealed before being cloned into pgRNA-*tetO*-JTetR (Addgene) by Golden Gate assembly. The plasmids pdCas9-bacteria (Addgene) and pgRNA-tetO-JTetR-H-NS were transformed into wildtype *Y*. *pseudotuberculosis* sequentially. These plasmids induce expression of dCas9 and gRNA-H-NS when exposed to anhydrotetracycline. *Y*. *pseudotuberculosis* cultures carrying these plasmids were sub-cultured to OD_600_ 0.2 and incubated at 26°C for 3 hrs in the presence or absence of 1μg/mL anhydrotetracycline, and then transferred to 37°C for 1.5 hrs to induce the T3SS. Samples were collected, and RNA was isolated for qRT-PCR analysis.

### Bioinformatic prediction of H-NS/YmoA binding sites

A training set of known H-NS binding sites in *E*. *coli* K-12 substr. MG1655 from RegulonDB was used to generate an H-NS binding motif using MEME-suite 5.1.1 tools [79,80]. FIMO was then used to scan for an H-NS binding site near the regulatory region of the *yscW-lcrF* promoter.

### ChIP-qPCR

Cells were grown for 3 hrs at 26°C or 37°C with shaking at 250 rpm and protein/nucleic acids were crosslinked using 1% formaldehyde at 26°C or 37°C for 10 min. Crosslinking was quenched with the addition of ice cold 0.1 M glycine and incubated at 4°C for 30 min. 32 x 1[OD_600_] cells were harvested for each replicate and cell pellets were stored at −80°C. DNA was fragmented by resuspending samples using IP buffer (100mM Tris-HCl, pH 8, 300mM NaCl, 1% Triton X-100, 1 mM PMSF) and sonicated at 25% Amplitude 15s on/59s off for a total of 8 cycles per sample. After sonication, lysates were treated with micrococcal nuclease and RNase-A for 1hr at 4°C. Lysates were clarified via centrifugation at 13,000 rpm for 15 min at 4°C. Lysates were pre-cleared using Dynabeads Protein A/G for 3hr at 4°C. Immunoprecipitation was performed by adding Sigma monoclonal mouse anti-FLAG M2 antibody to samples and incubated overnight at 4°C. Dynabeads Protein A/G were added to samples and washes were performed to remove non-specific binding. After H-NS-DNA or IscR-DNA complexes were eluted, samples were placed at 65°C for 5 hr to reverse crosslinks. DNA was then purified using Qiagen PCR purification kit and input samples were diluted 1:100 while samples treated with antibody or control samples not treated with the antibody were diluted 1:5 and qPCR was performed to assess IscR/H-NS binding to promoters of interest. Percent input was calculated by the following equation: 100*2^(CT^input^—CT^+AB^).

## Supporting information

S1 Text Fig A in S1 Text. 3xFLAG tag allows for detection of H-NS using FLAG antibody and does not affect H-NS ability to repress LcrF. Fig B in S1 Text. IscR does not regulate YmoA or H-NS expression. Fig C in S1 Text. IscR enrichment at the *suf* promoter is not influenced by temperature. Fig D in S1 Text. YmoA affects LcrF dependent type III secretion activity. Fig E in S1 Text. The YmoA^D43N^ mutant protein is expressed. Fig F in S1 Text. IscR binding to the *yscW-lcrF* promoter is dispensable in the absence of *ymoA*. Table A in S1 Text. Strains used in this study. Table B in S1 Text. *Y*. *pseudotuberculosis* primers used in this study. Table C in S1 Text. Plasmids used in this study.
(DOCX)
