# Supplementary material for: Repression by the H-NS/YmoA histone-like protein complex enables IscR dependent regulation of the Yersinia T3SS
Source: PLoS Genet. 2022 Jul 28;18(7):e1010321. doi: 10.1371/journal.pgen.1010321 (PMC9362927; doi:10.1371/journal.pgen.1010321)
Supplement: S1 Text — Fig A in S1 Text. 3xFLAG tag allows for detection of H-NS using FLAG antibody and does not affect H-NS ability to repress LcrF. Fig B in S1 Text. IscR does not regulate YmoA or H-NS expression. Fig C in S1 Text. IscR enrichment at the suf promoter is not influenced by temperature. Fig D in S1 Text. YmoA affects LcrF dependent type III secretion activity. Fig E in S1 Text. The YmoAD43N mutant protein is expressed. Fig F in S1 Text. IscR binding to the yscW-lcrF promoter is dispensable in the absence of ymoA. Table A in S1 Text. Strains used in this study. Table B in S1 Text. Y. pseudotuberculosis primers used in this study. Table C in S1 Text. Plasmids used in this study. (DOCX) [file pgen.1010321.s001.docx]

Supplementary Information for

**Repression by the H-NS/YmoA histone-like protein complex enables IscR dependent regulation of the *Yersinia* T3SS**

David Balderas^1^, Mané Ohanyan^1^, Pablo Alvarez^1^†, Erin Mettert^2^, Natasha Tanner^1^, Patricia J. Kiley^2^, and Victoria Auerbuch^1*^

^1^Department of Microbiology and Environmental Toxicology, University of California, Santa Cruz, Santa Cruz, California, USA

^2^ Department of Biomolecular Chemistry, University of Wisconsin-Madison, Madison, Wisconsin, USA

†Current Address: Department of Microbiology, Immunology & Molecular Genetics, University of California, Los Angeles, Los Angeles, California, USA

Victoria Auerbuch

Email: vastone@ucsc.edu

**This PDF file includes:**

Figures SA to SF

Table SA to SC

SI References


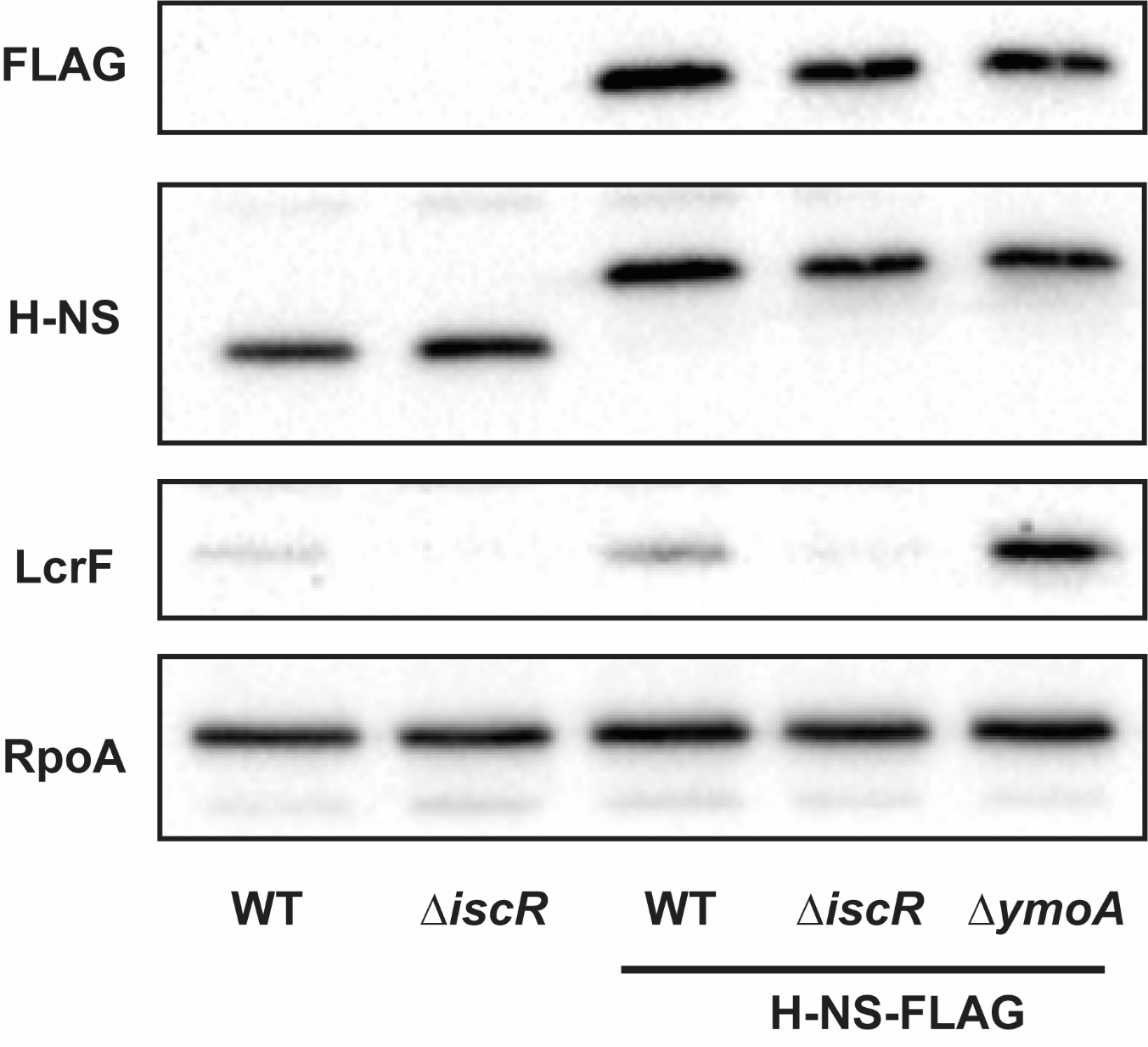


**Fig A. 3xFLAG tag allows for detection of H-NS using FLAG antibody and does not affect H-NS ability to repress LcrF.** Western blot analysis of whole cell extracts from WT *Y. pseudotuberculosis* or a strain harboring a chromosomally-encoded 3xFLAG tagged H-NS visualized using anti-FLAG, anti-HNS, anti-LcrF, or anti-RpoA antibodies. One representative experiment out of three biological replicates is shown.


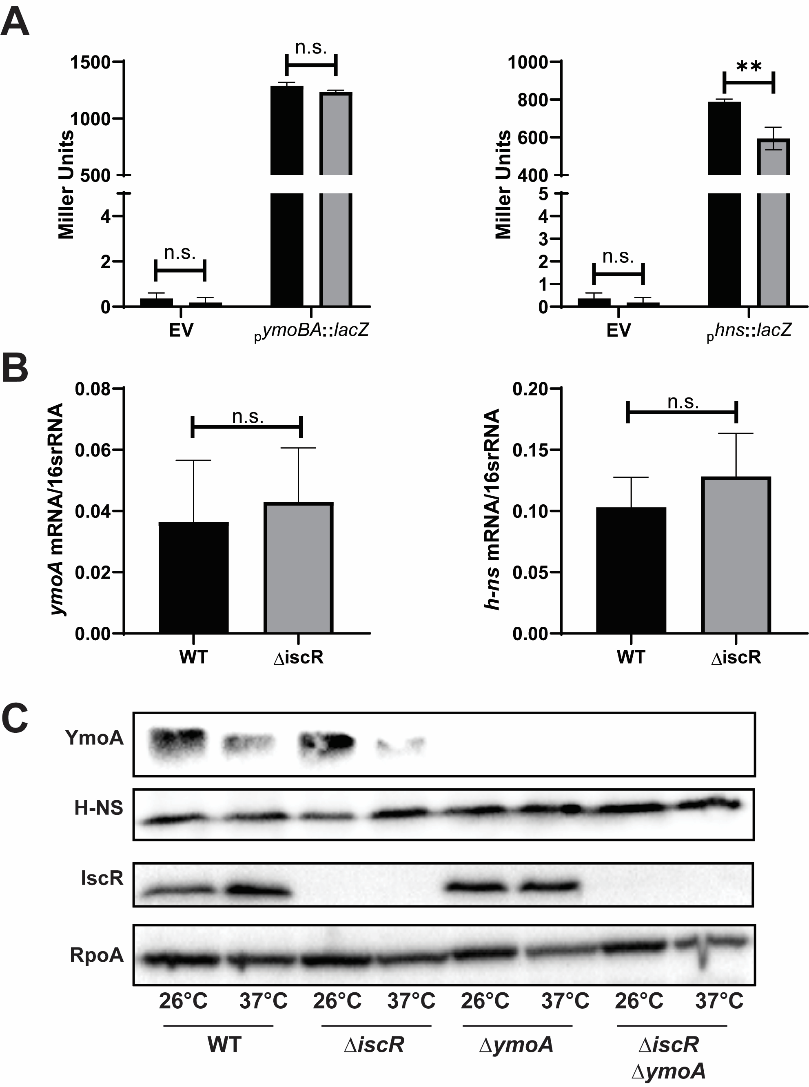


**Fig B. IscR does not regulate YmoA or H-NS expression. (A)** *Yersinia* strains harboring either the vector pFU99a (EV) or pFU99a plasmid encoding the *ymoBA* or *hns* promoters fused to *lacZ* were grown under T3SS inducing conditions and assayed for β-galactosidase activity (Miller units). Black bars represent the WT background and grey bars represent the *iscR* mutant background. The average of three biological replicates are shown ± standard deviation. (**B)** RNA was extracted from *Yersinia* strains grown under T3SS-inducing conditions and RT-qPCR was used to measure relative *ymoA* and *hns* mRNA levels normalized to 16S rRNA. The average of at least three biological replicates are shown ± standard deviation. **(C)** Western blot analysis of *Yersinia* strains grown in low calcium LB at 26°C or 37°C for 3 hours. Equal amounts of cell lysates were probed for RpoA, IscR, H-NS, and YmoA as indicated. One representative experiment out of three biological replicates is shown. Statistical analysis was performed using an unpaired Student’s t-test (n.s. non-significant).


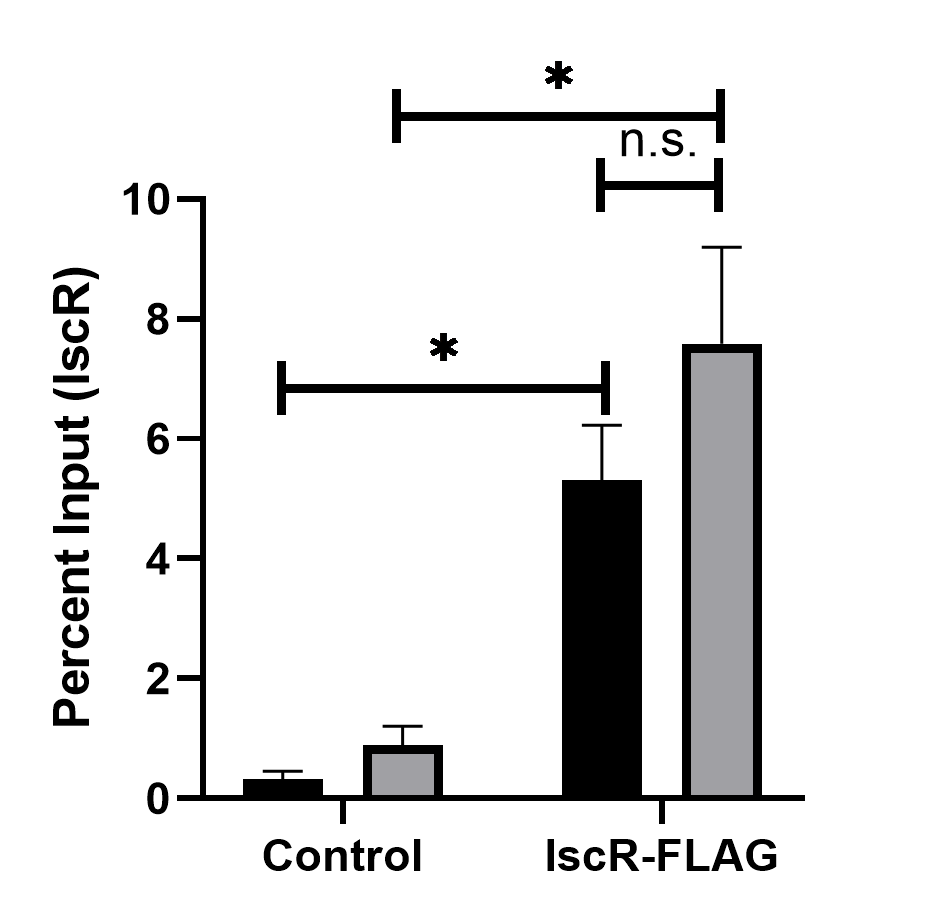


**Fig C. IscR enrichment at the *suf* promoter is not influenced by temperature.** The relative enrichment (percent input) of *suf* promoter DNA analyzed by ChIP-qPCR with the IscR-FLAG strain or the control strain (WT; non-FLAG tagged IscR). ChIP-qPCR was performed with bacteria grown at 26°C (black bars) or 37°C (grey bars). The average of three independent replicates ± standard deviation is shown and statistical analysis was performed using an unpaired Student’s t-test. (*p<.05, and n.s. non-significant).


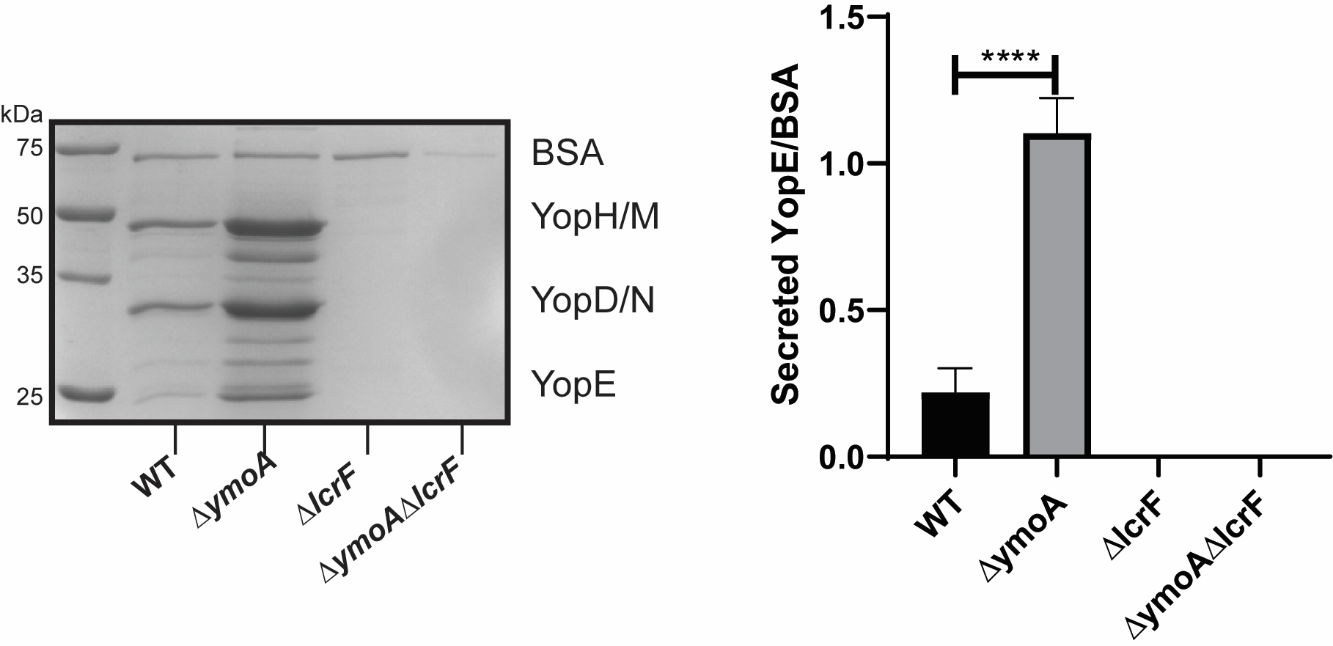


**Fig D. YmoA affects LcrF-dependent type III secretion activity.** *Yersinia* strains were grown in low calcium LB for 1.5 hrs at 26°C and transferred to 37°C (T3SS inducing conditions) for 1.5 hrs. The supernatant was collected and separated on a 12.5% SDS polyacrylamide gel, and subsequently stained with Coomassie blue (left panel). Bovine serum albumin (BSA) was used as a loading control. Gel bands were quantified by using Bio-Rad Image Lab Software Quantity and Analysis tools. YopE bands were normalized to the BSA loading control (right panel). The average of three independent replicates ± standard deviation is shown and statistical analysis was performed using an unpaired Student’s t-test. (****p<.0001).


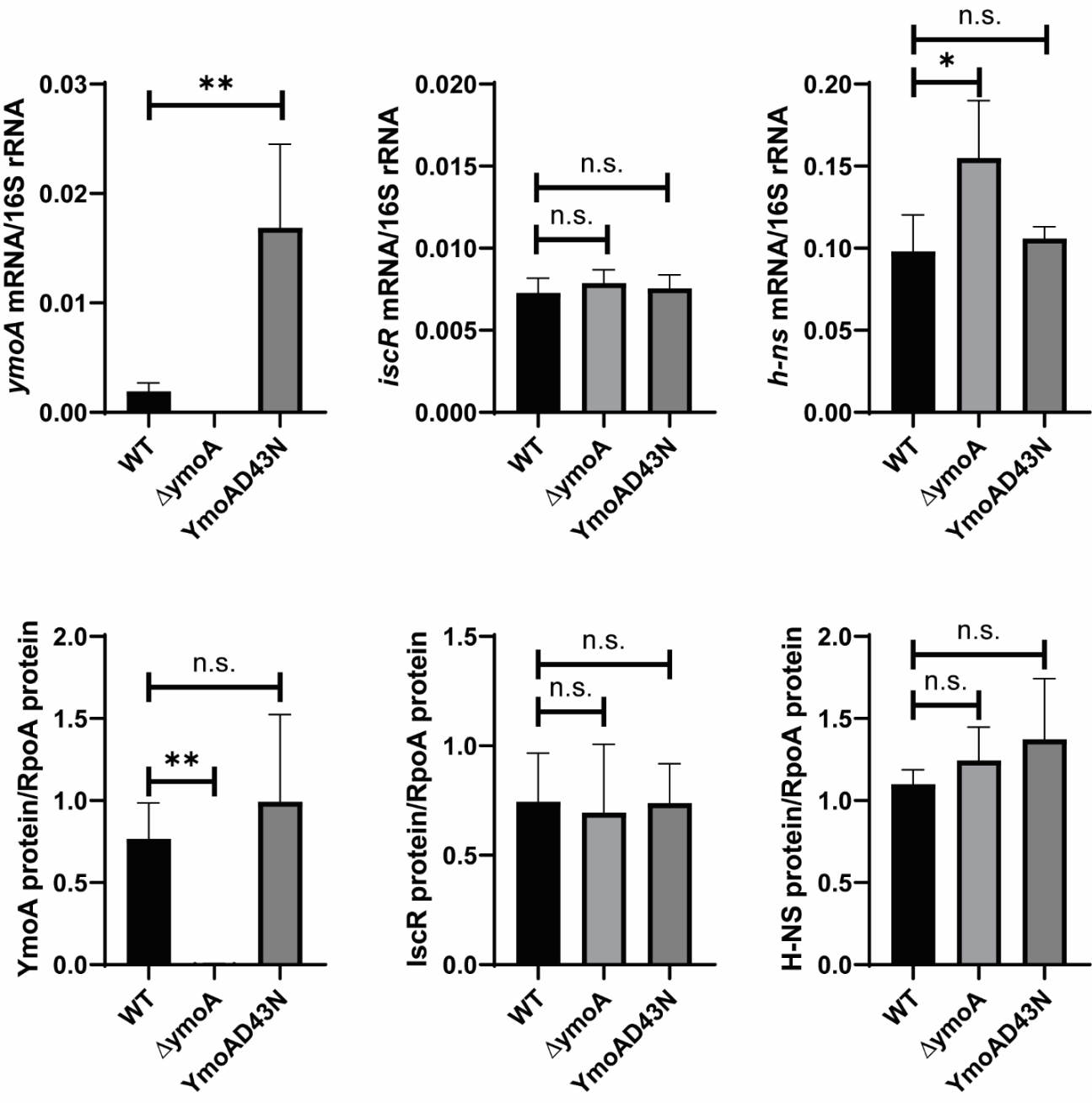


**Fig E. The YmoAD43N mutant protein is expressed*.*** RNA and whole cell extracts were prepared from indicated *Yersinia* strains grown in low calcium LB for 1.5 hrs at 26°C and transferred to 37°C (T3SS inducing conditions) for 1.5 hrs. *ymoA, iscR, and h-ns* mRNA expression levels was measured by RT-qPCR and normalized to 16S rRNA. The average of at least three biological replicates are shown with standard deviation (top panel). For western blots of cell extracts, proteins were visualized using anti-YmoA, anti-IscR, and anti-H-NS antibodies. Proteins were quantified by densitometry using BioRad Image Lab. The average of three biological replicates are shown with standard deviation (bottom panel). Statistical analysis was performed using an unpaired Student’s t-test. (*p<.05, **p<.01, and n.s. non-significant).

**

**

**Fig F. IscR binding to the *yscW-lcrF* promoter is dispensable in the absence of *ymoA.*** *Yersinia* strains were grown aerobically under T3SS-inducing conditions. **(a)** Levels of *lcrF* mRNA were measured and normalized to 16S rRNA using RT-qPCR. The average of at least three biological replicates are shown ± standard deviation. **(b)** LcrF protein levels were measured relative to the RpoA loading control by western blotting (left panel) and densitometry (right panel). Shown is the average of four biological replicates ± standard deviation. **(c)** Secreted proteins were precipitated and visualized by SDS-PAGE followed by Coomassie blue staining (left panel). The YopE bands were normalized to the BSA loading control (right panel). The average of three biological replicates ± standard deviations are shown. Statistical analysis was performed using an unpaired Student’s t-test (*p<.05, ***p < .001, and n.s. non-significant).

| **Table A. Strains used in this study.** | | |
| --- | --- | --- |
| **Strain** | **Relevant Genotype** | **Source or References** |
| IP2666/(WT) | Naturally lacks full-length YopT | [1] |
| IP2666/(Δ*iscR*) | *iscR* in frame deletion of codons 2 to 156 | [2] |
| IP2666/(IscR 3xFLAG) | In frame C-terminus 3xFLAG tag of chromosomal IscR | [3] |
| IP2666/(H-NS 3xFLAG) | In frame C-terminus 3xFLAG tag of chromosomal H-NS | This work |
| IP2666/(Δ*ymoA*) | *ymoA* in frame full deletion | [4] |
| IP2666/(Δ*iscR*Δ*ymoA*) | Double deletion mutant of *iscR* and *ymoA* | This work |
| IP2666/(*ymoA*^D43N^) | Single residue mutation of D43N YmoA | This work |
| IP2666/(Δ*iscR ymoA*^D43N^) | *iscR* in frame deletion in YmoA D43N mutant | This work |
| IP2666/(*lcrF*^pNull^) | Point mutations in IscR binding site upstream *yscW-lcrF* | [5] |
| IP2666/(Δ*ymoA* *lcrF*^pNull^) | *ymoA* in frame deletion in lcrF^pNull^ mutant | This work |
| IP2666/(Δ*lcrF*) | *lcrF* in frame full deletion | [6] |
| IP2666/(Δ*lcrF*Δ*ymoA*) | *ymoA* in frame deletion in *lcrF* mutant | This work |

| **Table B. *Y. pseudotuberculosis* primers used in this study.** | | |
| --- | --- | --- |
| **Name** | **Primer Sequence^a^** | **References** |
| qPCR_*16s*_F | AGCCAGCGGACCACATAAAG | [7] |
| qPCR_*16s*_R | AGTTGCAGACTCCAATCCGG | [7] |
| qPCR_*lcrF*_F | GGAGTGATTTTCCGTCAGTA | [2] |
| qPCR_*lcrF*_R | CTCCATAAATTTTTGCAACC | [2] |
| qPCR_*iscR*_F | CAGGGCGGAAATCGCTGCCT | [5] |
| qPCR_*iscR*_R | ATTAGCCGTTGCGGCGCCTAT | [5] |
| qPCR_*hns*_F | TGCAACAATACCGTGAAATG | This work |
| qPCR_*hns*_R | AGCACGTTTTGCTTTACCAG | This work |
| qPCR_*gyrA*_F | GGGGAAGTGGTGCTGAATAA | [8] |
| qPCR_*gyrA*_R | AAAATGGTACGGCGAGTCAC | [8] |
| qPCR_*cpxR*_F | TTGATGATGACCGTGAACTG | This work |
| qPCR_*cpxR*_R | ATCATAGGCGACCACAACAT | This work |
| qPCR_*rcsB*_F | GCAAATTGAATGGGTAAACG | This work |
| qPCR_*rcsB*_R | TAATTAGCACGTTGGCATCA | This work |
| qPCR_*ymoA*_F | CCTGATGCGTTTAAGAAAATG | This work |
| qPCR_*ymoA*_R | GATGGTCTGCAGCTGAGTAAA | This work |
| F*hns*_cds | cgaattcctgcagcccggggAGATGAGCACCATAAATG | This work |
| R*hns*_cds | aaccccccatCAACAGGAAGTCATCCAG | This work |
| F3xFLAG | cttcctgttgATGGGGGGTTCTGACTAC | This work |
| R3xFLAG | aataaaactaTCAACCTTTATCGTCGTCATC | This work |
| F3’*hns* | taaaggttgaTAGTTTTATTTCTTTAGCTATTACTATCG | This work |
| R3’*hns* | agggaacaaaagctggagctCGCCTAAATAGTCGTGGG | This work |
| F5’∆*ymoA* | cgaattcctgcagcccggggGATAGACAGCTGTATTTATATGAC | This work |
| R5’∆*ymoA* | gcgctaagcaGGTTTTTCTTCTCGATATACAAATTAATATTG | This work |
| F3’∆*ymoA* | aagaaaaaccTGCTTAGCGCTGGTTAAG | This work |
| R3’∆*ymoA* | agggaacaaaagctggagctCCTGTATTATCACTTTCCTGC | This work |
| pUC19-YmoA_F | acggccagtgaattcgagct*c*TTCATTTGTGATGAGTTTTAAAATAAAATAC | This work |
| pUC19-YmoA-R | cctgcaggtcgactctagag*gatcc*AGAGGGCTGAATTTGAATG | This work |
| *ymoA*^D43N^ _F | CTCAGCTGCAaACCATCGCCT | This work |
| *ymoA*^D43N^ _R | TAAAACAATTCCAGTTCATCATCAGAAAGTTCG | This work |
| pFU99a_*ymoA*_F | cctttcgtcttcacctcgagTAATTGGTATATTTTCAATGCTTGTTTGGATATCAATAC | This work |
| pFU99a_*ymoA*_R | ttcatttttaattcctcctgGTCATGCCGCTTAGGCGAG | This work |
| pFU99a_*hns*_F | cctttcgtcttcacctcgagATTGTACATAACGATACAGAAAC | This work |
| pFU99a_*hns*_R | ttcatttttaattcctcctgGTTGTTAAGAATTTTTAACGCTTC | This work |
| *hns*_gRNA_F | GCACTCCTAGTCTCAAATTATAAT | This work |
| *hns*_gRNA_R | AAACATTATAATTTGAGACTAGGA | This work |
| *hns*ChIP_site1_F | GCCCGTGCTCTTTATTGGG | This work |
| *hns*ChIP_site1_R | CACTTCAGCTGTGGCCTCTA | This work |
| *hns*ChIP_site2_F | TGGGGTGATTAACACCGG | This work |
| *hns*ChIP_site2_R | ATATAAGTGAACCTCTTGTTGGTTAAC | This work |
| *hns*ChIP_site3_F | TTATATGCGCAAGGTGTGATATTG | This work |
| *hns*ChIP_site3_R | TTCCCAATTATCTCAACGGGT | This work |
| *hns*ChIP_control_F | TGACGTCGGCAGTC | This work |
| *hns*ChIP_control_R | TCACCCCTTCGCAATAC | This work |
| *iscR*ChIP_*lcrF*_F | CGATATGGTTAACCAACAAGAGGTTC | This work |
| *iscR*ChIP_*lcrF*_R | GCACAGGAGAAATACAATTACCATAC | This work |
| *iscR*ChIP_*suf*_F | CTTTTAGACCTCCTTGGGTATCGC | This work |
| *iscR*ChIP_*suf*_R | CCGTTTGTTTTGCAGGGATATTAGG | This work |
| *iscR*ChIP_*hpt*_F | GCATGATGCTGGGCTTTAC | This work |
| *iscR*ChIP_*hpt*_R | ATAACAAAAATGCGCAGTGG | This work |
| pFU99a_*yscWlcrF*_R | ttcatttttaattcctcctgAGAAATGATGAGTGCTATAATACG | This work |
| pFU99a_*yscWlcrF*_p1 | cctttcgtcttcacctcgagCAAGTTCAGACTGTGCGC | This work |
| pFU99a_*yscWlcrF*_p2 | cctttcgtcttcacctcgagAGGCTGCAATGTAACTAG | This work |
| pFU99a_*yscWlcrF*_p3 | cctttcgtcttcacctcgagATGGTTAACCAACAAGAGG | This work |
| pFU99a_*yscWlcrF*_p4 | cctttcgtcttcacctcgagAATTAGGATTAATCTCTTGACTTTTTTTTG | This work |
| pFU99a_*yscWlcrF*_p5 | cctttcgtcttcacctcgagGGCTTTATATGCGCAAGG | This work |
| pFU99a p*yscW-lcrF*::*lacZ* Site 2_F | ccgttATATGGTTAACCAACAAGAG (Q5 mutagenisis) | This work |
| pFU99a p*yscW-lcrF*::*lacZ* Site 2_R | ccgcggCATATTCCCAATAGCCGG (Q5 mutagenisis) | This work |
| pFU99a p*yscW-lcrF*::*lacZ* Site 3_F | ccgttGTTCGTGGTGGACCCGTT (Q5 mutagenisis) | This work |
| pFU99a p*yscW-lcrF*::*lacZ* Site 3_R | ccgcggAGCTACTGCAATATCACACCTTG (Q5 mutagenisis) | This work |
| pFU99a p*yscW-lcrF*::*lacZ* pNull_F | gattTATACAGTATGGTAATTGTATTTC (Q5 mutagenisis) | This work |
| pFU99a p*yscW-lcrF*::*lacZ* pNull_R | ctttgAAATCGCATCATATATTCCTAATATAAG (Q5 mutagenisis) | This work |

^a^ Uppercase specifies primer that anneals to target for molecular cloning, lowercase is complementary sequence for NEB Gibson Assembly or extra nucleotides to facilitate efficient restriction digest

| **Table C. Plasmids used in this study.** | | |
| --- | --- | --- |
| **Name** | **Description** | **References** |
| pPK7179-*yscW-lcrF* | Promoter template for *in vitro* transcription, Amp^R^ | [3] |
| pPK7179-*sufA-*YP | Promoter template for *in vitro* transcription, Amp^R^ | [3] |
| pPK7179-*sufA-EC* | Promoter template for *in vitro* transcription, Amp^R^ | [9] |
| pSR47S Δ*ymoA* | Suicide vector for *ymoA* deletion, Kan^R^ | This work |
| pUC19 YmoA | Vector with CDS of YmoA, Amp^R^ | This work |
| pUC19 *ymoA*^D43N^ | Vector with CDS of YmoA with *ymoA*^D43N^ mutation, Amp^R^ | This work |
| pSR47S *ymoA*^D43N^ | Suicide vector for *ymoA*^D43N^ mutation, Kan^R^ | This work |
| pdCas9-bacteria | Expressing dCas9 protein under the control of an ATc-inducible promoter with a TetR casette, Cm^R^ | [10] |
| pgRNA-tetO-JTetR | Expressing TetR driven by promoter J23119 and sgRNA driven by PL2tetO. Amp^R^ | [11] |
| pgRNA-tetO-JTetR-H-NS | 20-bp targeting sequence for *hns* gene was inserted into pgRNA-tetO-JTetR, Amp^R^ | This work |
| pFU99a | Empty vector carrying promoter-less *lacZ* fusion, Cm^R^ | This work |
| pFU99a p*ymoBA*::*lacZ* | The promoter of *ymoBA* fused to *lacZ*, Cm^R^ | This work |
| pFU99a p*hns*::*lacZ* | The promoter of *hns* fused to *lacZ*, Cm^R^ | This work |
| pFU99a p*yscW-lcrF*::*lacZ* p1 | The promoter of *yscW-lcrF* (-505 - +294) fused to *lacZ*, Cm^R^ | This work |
| pFU99a p*yscW-lcrF*::*lacZ* p2 | The promoter of *yscW-lcrF* (-309 - +294) fused to *lacZ*, Cm^R^ | This work |
| pFU99a p*yscW-lcrF*::*lacZ* p3 | The promoter of *yscW-lcrF* (-166 - +294) fused to *lacZ*, Cm^R^ | This work |
| pFU99a p*yscW-lcrF*::*lacZ* p4 | The promoter of *yscW-lcrF* (-47 - +294) fused to *lacZ*, Cm^R^ | This work |
| pFU99a p*yscW-lcrF*::*lacZ* p5 | The promoter of *yscW-lcrF* (+101 - +294) fused to *lacZ*, Cm^R^ | This work |
| pFU99a p*yscW-lcrF*::*lacZ* Site 2 | The promoter of *yscW-lcrF* (-309 - +294) with a mutation in HNS binding site 2 fused to *lacZ*, Cm^R^ | This work |
| pFU99a p*yscW-lcrF*::*lacZ* Site 3 | The promoter of *yscW-lcrF* (-309 - +294) with a mutation in HNS binding site 3 fused to *lacZ*, Cm^R^ | This work |
| pFU99a p*yscW-lcrF*::*lacZ* Site2_Site 3 | The promoter of *yscW-lcrF* (-309 - +294) with a mutation in HNS binding site 2 and site 3 fused to *lacZ*, Cm^R^ | This work |
| pFU99a p*yscW-lcrF*::*lacZ* pNull | The promoter of *yscW-lcrF* (-309 - +294) with a mutation in the IscR binding site fused to *lacZ*, Cm^R^ | This work |
| pFU99a p*yscW-lcrF*::*lacZ* pNull_Site 2 | The promoter of *yscW-lcrF* (-309 - +294) with a mutation in the IscR binding site and HNS binding site 2 fused to *lacZ*, Cm^R^ | This work |
| pFU99a p*yscW-lcrF*::*lacZ* pNull_Site 3 | The promoter of *yscW-lcrF* (-309 - +294) with a mutation in the IscR binding site and HNS binding site 3 fused to *lacZ*, Cm^R^ | This work |
| pFU99a p*yscW-lcrF*::*lacZ* pNull_Site2_Site 3 | The promoter of *yscW-lcrF* (-309 - +294) with a mutation in the IscR binding site and HNS binding site 2 and site 3 fused to *lacZ*, Cm^R^ | This work |

References:

1. Bliska JB, Guan K, Dixon JE, Falkow S. Tyrosine phosphate hydrolysis of host proteins by an essential *Yersinia* virulence determinant. Proc Natl Acad Sci U S A. 1991. doi:10.1073/pnas.88.4.1187

2. Miller HK, Kwuan L, Schwiesow L, Bernick DL, Mettert E, Ramirez HA, et al. IscR Is Essential for *Yersinia pseudotuberculosis* Type III Secretion and Virulence. PLoS Pathog. 2014. doi:10.1371/journal.ppat.1004194

3. Balderas D, Mettert E, Lam HN, Banerjee R, Gverzdys T, Alvarez P, et al. Genome Scale Analysis Reveals IscR Directly and Indirectly Regulates Virulence Factor Genes in Pathogenic *Yersinia*. MBio. 2021;12: e00633-21.

4. Böhme K, Steinmann R, Kortmann J, Seekircher S, Heroven AK, Berger E, et al. Concerted actions of a thermo-labile regulator and a unique intergenic RNA thermosensor control *Yersinia* virulence. PLoS Pathog. 2012. doi:10.1371/journal.ppat.1002518

5. Hooker-Romero D, Mettert E, Schwiesow L, Balderas D, Alvarez PA, Kicin A, et al. Iron availability and oxygen tension regulate the *Yersinia* Ysc type III secretion system to enable disseminated infection. PLoS Pathog. 2019. doi:10.1371/journal.ppat.1008001

6. Garrity-Ryan LK, Kim OK, Balada-Llasat JM, Bartlett VJ, Verma AK, Fisher ML, et al. Small molecule inhibitors of LcrF, a *Yersinia pseudotuberculosis* transcription factor, attenuate virulence and limit infection in a murine pneumonia model. Infect Immun. 2010. doi:10.1128/IAI.01305-09

7. Yang Y, Merriam JJ, Mueller JP, Isberg RR. The psa locus is responsible for thermoinducible binding of *Yersinia pseudotuberculosis* to cultured cells. Infect Immun. 1996. doi:10.1128/iai.64.7.2483-2489.1996

8. Davis KM, Mohammadi S, Isberg RR. Community Behavior and Spatial Regulation within a Bacterial Microcolony in Deep Tissue Sites Serves to Protect against Host Attack. Cell Host Microbe. 2015. doi:10.1016/j.chom.2014.11.008

9. Giel JL, Rodionov D, Liu M, Blattner FR, Kiley PJ. IscR-dependent gene expression links iron-sulphur cluster assembly to the control of O2-regulated genes in *Escherichia coli*. Mol Microbiol. 2006. doi:10.1111/j.1365-2958.2006.05160.x

10. Qi LS, Larson MH, Gilbert LA, Doudna JA, Weissman JS, Arkin AP, et al. Repurposing CRISPR as an RNA-guided platform for sequence-specific control of gene expression. Cell. 2013. doi:10.1016/j.cell.2013.02.022

11. Wang T, Wang M, Zhang Q, Cao S, Li X, Qi Z, et al. Reversible gene expression control in *Yersinia pestis* by using an optimized CRISPR interference system. Appl Environ Microbiol. 2019. doi:10.1128/AEM.00097-19
